# A genomics-led approach to deciphering the mechanism of thiotetronate antibiotic biosynthesis†
†Electronic supplementary information (ESI) available: Fig. S1–S21; Tables S1–S5, full experimental details and procedures. See DOI: 10.1039/c5sc03059e



**DOI:** 10.1039/c5sc03059e

**Published:** 2015-10-08

**Authors:** W. Tao, M. E. Yurkovich, S. Wen, K. E. Lebe, M. Samborskyy, Y. Liu, A. Yang, Y. Liu, Y. Ju, Z. Deng, M. Tosin, Y. Sun, P. F. Leadlay

**Affiliations:** a Key Laboratory of Combinatorial Biosynthesis and Drug Discovery (Wuhan University) , Ministry of Education , Wuhan University School of Pharmaceutical Sciences , Wuhan 430071 , People's Republic of China . Email: yhsun@whu.edu.cn; b Department of Biochemistry , University of Cambridge , Sanger Building, 80 Tennis Court Road , Cambridge CB2 1GA , UK . Email: pfl10@cam.ac.uk; c Department of Chemistry , University of Warwick , Library Road , Coventry CV4 7AL , UK

## Abstract

Thiolactomycin (TLM) is a thiotetronate antibiotic that selectively targets bacterial fatty acid biosynthesis through inhibition of the β-ketoacyl-acyl carrier protein synthases (KASI/II) that catalyse chain elongation on the type II (dissociated) fatty acid synthase. It has proved effective in *in vivo* infection models of *Mycobacterium tuberculosis* and continues to attract interest as a template for drug discovery. We have used a comparative genomics approach to uncover the (hitherto elusive) biosynthetic pathway to TLM and related thiotetronates. Analysis of the whole-genome sequence of *Streptomyces olivaceus* Tü 3010 producing the more ramified thiotetronate Tü 3010 provided initial evidence that such thiotetronates are assembled by a novel iterative polyketide synthase-nonribosomal peptide synthetase, and revealed the identity of other pathway enzymes, encoded by adjacent genes. Subsequent genome sequencing of three other thiotetronate-producing actinomycetes, including the *Lentzea* sp. ATCC 31319 that produces TLM, confirmed that near-identical clusters were also present in these genomes. In-frame gene deletion within the cluster for Tü 3010 from *Streptomyces thiolactonus* NRRL 15439, or within the TLM cluster, led to loss of production of the respective thiotetronate, confirming their identity. Each cluster houses at least one gene encoding a KASI/II enzyme, suggesting plausible mechanisms for self-resistance. A separate genetic locus encodes a cysteine desulfurase and a (thiouridylase-like) sulfur transferase to supply the sulfur atom for thiotetronate ring formation. Transfer of the main Tü 3010 gene cluster (*stu* gene cluster) into *Streptomyces avermitilis* led to heterologous production of this thiotetronate, showing that an equivalent sulfur donor can be supplied by this host strain. Mutational analysis of the Tü 3010 and TLM clusters has revealed the unexpected role of a cytochrome P450 enzyme in thiotetronate ring formation. These insights have allowed us to propose a mechanism for sulfur insertion, and have opened the way to engineering of the biosynthesis of TLM and other thiotetronates to produce novel analogues.

## Abstract 2

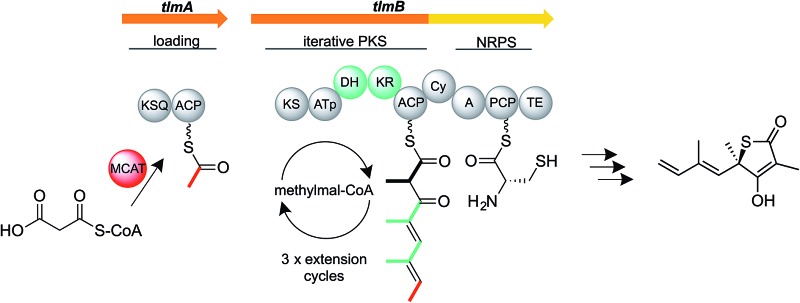
A novel mechanism is proposed for ring formation in the biosynthetic pathway to thiotetronate antibiotics thiolactomycin and Tü 3010.

## Introduction

The advent of rapid next-generation DNA sequencing has revolutionized the search for chemical diversity from antibiotic-producing micro-organisms. Actinomycete genomes (6–12 Mbp) have been revealed to encode very large numbers of biosynthetic gene clusters,^1,2^ and sophisticated bioinformatic tools are available to assist the assignment of the products of each encoded pathway to particular structural classes.^3^ The link between the sequence of the genes and the chemical structure of the product is particularly clear for pathways utilizing giant assembly-line synthases to produce complex reduced polyketides or nonribosomally-determined peptides.^4,5^ However, there remain a number of valuable chemotypes whose biosynthetic pathways are obscure, and also many orphan clusters whose potentially novel products remain to be elucidated. Methods for systematic analysis of the entire complement of secondary metabolite biosynthetic gene clusters in a strain remain cumbersome and slow. We describe here an efficacious way of targeting a sought-after biosynthetic pathway, by sequencing several strains producing compounds from the same structural class. This effectively harnesses the accumulated knowledge from decades of conventional natural product screening and structure determination, and significantly reduces the time taken to identify and validate the involvement of the genes and enzymes concerned. We have used this approach to uncover the biosynthetic pathway to thiotetronate antibiotics, revealing the unexpected role of an iterative polyketide synthase-nonribosomal peptide synthetase and gaining insight into both sulfur insertion and the mechanism of self-resistance.

**Fig. 1:**
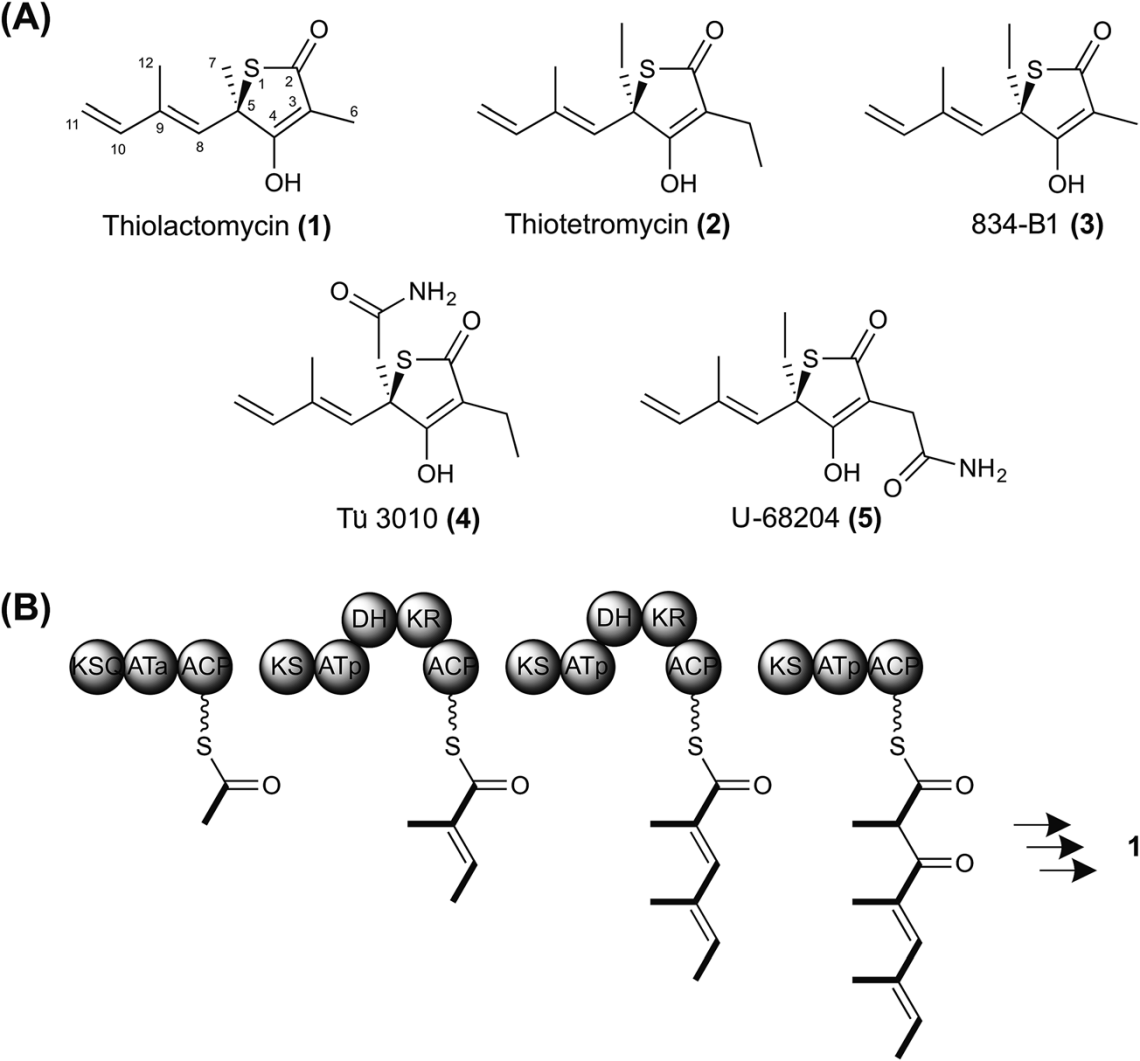
Thiotetronate biosynthesis. (A) Reported structures of naturally-occurring thiotetronates: thiolactomycin (**1**), thiotetromycin (**2**), 834-B1 (**3**), Tü 3010 (**4**), and U-68204 (**5**); (B) hypothetical scheme for thiolactomycin biosynthesis, assuming the operation of a trimodular polyketide synthase. Sulfur insertion has been proposed to involve either a thiirane intermediate or a perthioester.^21^

Thiolactomycin ((TLM) (**1**) Fig. 1A) is a thiolactone natural product originally isolated from a soil *Nocardia* strain that has since been re-named as *Lentzea* sp. ATCC 31319.^6,7^ Its configuration was subsequently established as the (5*R*)-isomer by total synthesis.^8^ It is a reversible inhibitor of the β-ketoacyl-acyl carrier protein synthase (KAS) enzymes of the bacterial type II (dissociated) fatty acid synthase (FAS)^9–11^ by binding preferentially to the acyl-enzyme intermediate.^10,12^ It shows broad antibiotic activity against both Gram-positive and Gram-negative bacteria, and has been shown effective in murine models of infection.^13^ The type II FAS condensing enzyme KasA in *Mycobacterium tuberculosis* is essential for cell wall mycolic acid biosynthesis and has been validated as an attractive drug target.^14–17^ TLM has also shown encouraging anti-malarial and anti-trypanosomal activity through its inhibition of apicoplast type II FAS^18^ and has served as a platform for development of antimalarial drugs.^19^ Meanwhile, synthetic TLM analogs have been devised that (unlike the parent molecule) inhibit human fatty acid synthase, an emerging target in combatting obesity as well as certain cancers.^20^ Despite this evidence for intriguing therapeutic activity, almost nothing is known of TLM biosynthesis. Importantly, Reynolds and colleagues have shown,^21^ by feeding isotopically-labeled precursors, that TLM is produced by a polyketide biosynthetic pathway, and also that the sulfur atom of the thiolactone may derive from cysteine. They proposed a mechanism in which a linear dienoyl tetraketide thioester is assembled on a multimodular polyketide synthase (PKS) (Fig. 1B) and suggested two alternative mechanisms for sulfur insertion.^21^ However, no progress has been reported since then.

We have undertaken the deciphering of the pathway to TLM, as well as the pathway to closely-related thiotetronates (Fig. 1). Of these, thiotetromycin (**2**) was discovered in 1983 from *Streptomyces* sp. OM-674 and differs structurally from TLM only in the incorporation of two butyrate extender units instead of propionate units,^22^ while *Streptomyces* sp. Y-0834H produces, in addition to (**1**) and (**2**), compound 834-B1 (**3**) containing only one butyrate extender unit.^23^ The compound Tü 3010 (**4**) from *Streptomyces olivaceus* Tü 3010 apparently represents a further processing of thiotetromycin, in which the ethyl group at C-5 of the lactone ring is oxidised first to a primary alcohol, then to the corresponding carboxylic acid, and finally converted to a carboxamide.^24^ *Streptomyces thiolactonus* NRRL 15439 ^25^ and *Streptomyces* sp. MG11, a strain isolated in Ghana,^26^ both produce a thiotetronate which was originally identified as U-68204 (**5**), a positional isomer of Tü 3010 in which the carboxamidomethyl group is attached instead at C-3 of the thiolactone ring.^25,26^ Using 1D- and 2D-NMR, we have re-analysed the structure of this thiotetronate, purified both from *S. thiolactonus* NRRL 15439 and *Streptomyces* sp. MG11. This analysis has shown that U-68204 is actually identical to Tü 3010 (**4**) (Fig. S1–S3, and Table S1†). Of the known thiotetronates related to TLM, Tü 3010 is reported to be 15-fold more effective than the others as an antibacterial *in vivo*.^27^

When an initial genome sequence scan of the TLM producer failed to reveal a plausible candidate gene cluster encoding the expected trimodular assembly-line PKS (Fig. 1B), we turned to analyse the Tü 3010 cluster in *S. olivaceus* Tü 3010, arguing that the additional functional group and more ramified structure would aid identification of a novel type of cluster organization. Our results have vindicated this approach and now enable us to make a mechanistic proposal for thiotetronate biosynthesis that opens the way to exploiting the pathway for drug discovery, as well as revealing new aspects of the poorly-understood enzymology of sulfur incorporation into secondary metabolites.

## Results and discussion

### Identification and *in silico* analysis of a unique iterative type I PKS gene cluster in *S. olivaceus* Tü 3010

The whole-genome sequence of *S. olivaceus* Tü 3010 (9.7 Mbp) was determined by a combination of shotgun and long-range mate-pair Illumina sequencing, assembled using an in-house pipeline into eight scaffolds, and displayed using the program Artemis.^28^ On the assumption that the carboxamide function of Tü 3010 is produced from the corresponding carboxylic acid by an enzyme resembling the amide synthetase of rimocidin biosynthesis^29^ the sequence was screened in a BLASTp search^30^ using this protein sequence as a probe. Of the three significant hits, one gene with high similarity to authentic ATP-dependent asparagine synthases proved to be clustered together with an unusual polyketide synthase (PKS) and nonribosomal peptide synthetase (NRPS) multienzyme, and on further analysis (see below) this locus was identified as a strong candidate for the Tü 3010 biosynthetic gene cluster.

**Fig. 2:**
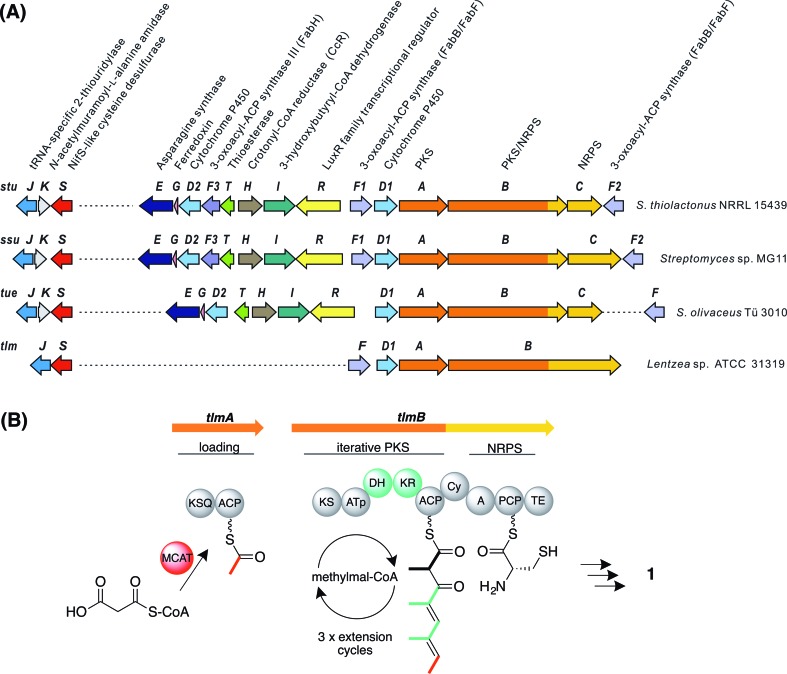
Genes and enzymes involved in thiotetronate biosynthesis. (A) Gene organisation of sequenced thiotetronate biosynthetic gene clusters; *stu*, *ssu*, *tue*, and *tlm* (Table S2†). A separate locus, containing a specific cysteine desulfurase and thiouridylase, is required for sulfur insertion; (B) domain organisation of the iterative PKS multienzyme proposed to assemble the tetraketide backbone of thiolactomycin. The initiating PKS encoded by *tlmA* contains only a KSQ and an ACP domain, so the acyltransferase ATa of the loading module is likely contributed by FAS malonyl-CoA:ACP acyltransferase (MCAT). The single PKS module within *tlmB* catalyses all three cycles of chain elongation, incorporating methylmalonyl-CoA at each round. The reductive domains KR and DH are only active in the first and second extension rounds, as indicated in green. Also within *tlmB* are NRPS-related Cy, A and PCP domains, and a TE domain. The A domain is predicted to activate l-cysteine. In the *tue, stu*, and *ssu* clusters the A and PCP domains are located on a separate ORF. KSQ, malonyl-CoA decarboxylase; ATp, methylmalonyl-CoA-specific acyltransferase; KS, ketosynthase; KR, ketoreductase; DH, dehydratase; ACP, acyl carrier protein; Cy, cyclisation; A, adenylation; PCP, peptide carrier protein; TE, thioesterase.

Three open reading frames (ORFs) designated *tueA*, *tueB*, and *tueC* appear to participate in assembling the tetraketide backbone. Flanking these genes, another eight ORFs are potentially involved in the biosynthetic pathway (Fig. 2A and Table S2†). TueA contains an initiation module comprising an N-terminal KSQ domain^31^ and an acyl carrier protein (ACP) domain and is proposed to catalyse the attachment of a malonyl group to the ACP and its subsequent decarboxylation to provide the acetate starter unit for Tü 3010. However this loading module lacks the required acyltransferase (AT) domain and we propose that this function may be supplied by the malonyl-CoA:ACP acyltransferase of fatty acid biosynthesis (MCAT). TueB is a novel multienzyme containing a single PKS extension module with the predicted domain order from the N-terminus: ketosynthase (KS) – acyltransferase (AT) – dehydratase (DH) – ketoreductase (KR) – ACP. The specificity motif of the AT (CASH) differs from that expected for recruitment of either malonyl extender units (HAFH) or methylmalonyl extender units (YASH), but is consistent with incorporation of an ethylmalonyl unit (Fig. S4†).^32–34^ The tetraketide backbone of Tü 3010 is assembled by an unexpected iterative PKS, recruiting a propionate unit in the first chain extension cycle and then two butyrate units (Fig. S5†). TueB also houses a C-terminal domain bearing significant sequence resemblance to the cyclisation (Cy) domains of NRPS multienzymes, which typically catalyse the heterocyclisation of cysteine and serine/threonine to thiazoline and oxazoline rings.^35^ This TueB domain contains the active site sequence motif DxxxxDxxS (where x is any amino acid) conserved in Cy domains.^36^ TueC contains an adenylation domain predicted to be specific for the activation of l-cysteine, and a peptidyl carrier protein (PCP) domain to which the cysteinyl residue is proposed to become tethered.^37^ The co-location of a gene encoding cysteine activation with an unusual PKS pointed to the identity of this cluster as governing thiotetronate biosynthesis. Further support for this hypothesis came from examination of flanking genes, which include two putative cytochrome P450 genes (*tueD1* and *tueD2*) and the gene for an asparagine synthase-like enzyme (*tueE*), which would use ATP and glutamine as the amino donor, to provide the carboxamido group found in Tü 3010. The cluster houses genes for 3-hydroxybutyryl-CoA dehydrogenase (*tueI*) and for a carboxylating enoyl-thioester reductase (*tueH*) bolstering the identification of the cluster as governing production of a butyrate unit-containing polyketide.^38^ The cluster also contains putative regulatory genes, and a gene for a stand-alone thioesterase (TE) (*tueT*) which may assist in aberrant chain removal from the PKS.^39^

### Identification of polyketide synthase gene clusters for biosynthesis of thiolactomycin and other thiotetronates

Using as probes individual genes from the *tue* gene cluster for Tü 3010, BLASTp searches then were used to identify closely homologous gene clusters in the whole-genome sequences of *Lentzea* sp. ATCC 31319 (10.5 Mbp), producing (**1**); *S. thiolactonus* NRRL 15439 (9.6 Mbp) producing Tü 3010 (**4**) (originally reported^25^ to produce (**5**)); and *Streptomyces* sp. MG11 (9.7 Mbp) also producing (**4**). The organisation of these clusters is shown in Fig. 2A and the proposed function of each ORF is given in Table S2.† The simplest of these homologous clusters is that for TLM, which houses an iterative PKS (*tlmA*, *tlmB*) with exactly the complement of domains seen for Tü 3010 except that the AT domain of the single extension module has the specificity motif YASH, as predicted for incorporation of methylmalonyl units in all three cycles of polyketide chain extension (Fig. 2B).^34^ Additionally, the ORFs *tueB* and *tueC* are here fused into a single ORF *tlmB*; and there is a C-terminal thioesterase (TE) domain in TlmB, rather than a discrete TEII enzyme as in the Tü 3010 cluster.

All the genes in Tü 3010 interpreted as required for post-PKS oxidative processing, or for the furnishing of butyrate extender units, are missing from the candidate *tlm* cluster, except for a single cytochrome P450, *tlmD1*. Tellingly, this cluster also contains a gene, *tlmF,* encoding a KASI/II enzyme (FabB/FabF), which plays an essential role in fatty acid biosynthesis and is the known intracellular target of TLM and related thiolactones. The significance of this is underscored by the gene organisation of the biosynthetic clusters for the same thiotetronate (**4**) obtained from two other strains (Fig. 2A), each of which shows that there is one *fabH* and two *fabB/fabF-*related genes found in the cluster. These findings immediately suggest plausible mechanisms by which these strains become resistant to the effects of their own antibiotic. The cluster-encoded copies of FabB/FabF and FabH may differ sufficiently in their active sites from wild-type KASI/II enzymes that they are not inhibited by thiotetronate; and/or the additional copies may ensure a high level of KASI/II activity that overcomes the effects of the (reversible) inhibition by thiolactone antibiotics (Fig. S6 and S7†).^40–44^ Further work will be required to deconvolute these effects. Within 21 kbp of the *tue* cluster, there is a gene encoding an additional FabB/FabF-like KASI/II enzyme, *tueF* (Fig. 2A, Table S2†), which may play an analogous role in the biosynthesis of Tü 3010.

In addition to revealing plausible candidates for the biosynthetic gene clusters for thiotetronates (**1**) and (**4**), a search of published genome sequences using the bioinformatics tool antiSMASH^3^ revealed a gene cluster similar to that for TLM in the fully-sequenced genome of *Streptomyces cattleya* NRRL 8057 (SCAT5757-SCAT5761).^45^ Manual BLAST searching uncovered a further two Tü 3010-like gene clusters in, respectively, *Streptomyces afghanensis* 277 (NCBI identifiers EPJ35918 to EPJ34243) and *Streptomyces incarnatus* NRRL 8089 (NCBI identifiers ABB07_06605 to ABB07_06685). Since a thiotetronate has not previously been reported to be produced by these strains, the clusters may be cryptic under laboratory conditions.

### Genetic confirmation of the involvement of the *stu* gene cluster in Tü 3010 biosynthesis, and of the *tlm* gene cluster in thiolactomycin biosynthesis

To confirm the link between the putative thiotetronate clusters and the production of the relevant thiotetronate antibiotics, individual genes were inactivated both in *S. thiolactonus* NRRL 15439 and *Lentzea* sp. ATCC 31319. In *S. thiolactonus*, in-frame deletions were introduced into the cluster-associated *stuH*, a gene encoding a carboxylating enoyl-thioester reductase (*ccr*) (Fig. S8†); and also into *stuB*, the gene encoding the iterative PKS-NRPS (Fig. S9†). LC-ESI-HRMS analysis of the ΔstuH mutant strain showed that production of Tü 3010 was reduced to only 1% of wild-type levels, supporting its identification as an important supplier of ethylmalonyl-CoA extender units derived from crotonyl-CoA. In the ΔstuB mutant strain of Tü 3010 biosynthesis was completely abolished, confirming the role for this multienzyme in thiotetronate biosynthesis.

Similarly, an in-frame deletion was created in the PKS-NRPS gene *tlmA* in order to link the putative *tlm* cluster in *Lentzea* sp. ATCC 31319 to the production of TLM. Analysis of the ΔtlmA mutant strain using LC-ESI-MS showed that TLM production had indeed been abolished (Fig. S10 and S11†). When a copy of the *tlmA* gene was used to complement^46^ *Lentzea* sp. ΔtlmA *in trans*, LC-ESI-MS analysis of strain *Lentzea* sp. ΔtlmA::pIB-tlmA showed TLM production was restored to approximately 70% of wild-type levels confirming that the *tlm* locus is indeed responsible for TLM biosynthesis (Fig. S11†).

### Enzymes governing insertion of sulfur into thiotetronate Tü 3010 are hijacked from primary metabolism

Previous work has provided preliminary evidence that the sulfur atom in TLM is derived from cysteine,^21^ the central metabolite of sulfur biochemistry in prokaryotic cells.^47^ Pyridoxal phosphate-dependent cysteine desulfurases separate the sulfur atom from cysteine in the form of persulfidic sulfur (R-S-SH).^48,49^ The activated sulfur is utilized in primary metabolism in the biosynthesis of iron sulfur clusters, cofactors such as thiamin, molybdopterin, biotin and lipoic acid, and the thio modification of tRNA.^50^ None of the four candidate thiotetronate clusters we have uncovered contains enzymatic machinery for sulfur insertion, so those enzymes are encoded elsewhere on the genome, and may indeed form part of the sulfur insertion machinery of primary metabolism.^51^

**Fig. 3:**
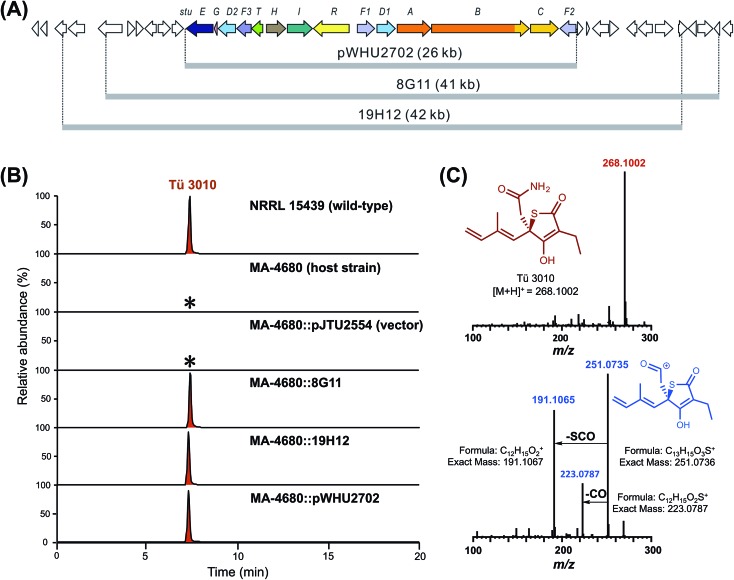
Heterologous expression of Tü 3010 biosynthetic genes in *S. avermitilis* MA-4680. (A) Organisation of the proposed *stu* cluster in *S. thiolactonus* NRRL 15439 library cosmids 8G11 and 19H12, and in the truncated insert derived from 8G11; (B) LC-ESI-HRMS selective ion trace of Tü 3010. The asterisk means not detected; (C) the mass confirmation of Tü 3010 production by MS–MS analysis. The major MS–MS fragments of [M + H]^+^ (*m*/*z* 268.1002) in the heterologous host strains MA-4680::8G11, MA-4680::19H12, and MA-4680::pWHU2702 are loss of ammonia (NH_3_), carbonyl sulfide (SCO), and carbon monoxide (CO), which is identical to the pattern seen in the known producer *S. thiolactonus* NRRL 15439, confirming heterologous Tü 3010 production.

We identified a set of three contiguous genes (*stuJ*, *stuK*, and *stuS* in *S. thiolactonus* NRRL 15439) almost fully conserved in the four genomes (Fig. 2A), that encode respectively a sulfur transferase (annotated as a tRNA-specific 2-thiouridylase), an *N*-acetylmuramoyl-l-alanine amidase and a NifS-like cysteine desulfurase (the amidase is missing in the *Lentzea* sp. ATCC 31319). We then carried out gene deletions in this region in *S. thiolactonus* to determine the contribution of these genes to Tü 3010 biosynthesis (Fig. S12–S15†). When all three genes were deleted, the resulting triple mutant grew normally but LC-ESI-HRMS analysis showed the complete loss of Tü 3010 production. Likewise, loss of the cysteine desulfurase gene *stuS* alone abolished Tü 3010 production, while *stuK* had no effect on biosynthesis, suggesting that only the desulfurase and the 2-thiouridylase product of *stuJ* are involved in Tü 3010 biosynthesis. In agreement with this, *in trans* complementation of the triple mutant with the cysteine desulfurase gene *stuS* alone gave 20% of wild-type levels of Tü 3010, and complementation with *stuJ* and *stuS* together was required to fully restore production. Homologues of *stuJ* and *stuS* are present in most sequenced *Streptomyces* genomes, including those of model organisms *Streptomyces lividans*, *Streptomyces avermitilis* and *Streptomyces coelicolor* suggesting that they play additional roles in primary metabolism.^48^ We therefore wished to test whether, if the *stu* gene cluster were transplanted into one of these heterologous hosts, the resident sulfur insertion apparatus of the host would support Tü 3010 biosynthesis. This would allow the limits of the *stu* cluster to be more precisely defined, and would speed up genetic manipulation of the Tü 3010 pathway. The Tü 3010 biosynthetic gene cluster was cloned into the heterologous host strain *S. avermitilis* MA-4680. Two independent cosmids, 8G11 and 19H12 (Fig. 3) whose inserts comfortably encompass the annotated *stu* cluster, were separately introduced into the host by conjugation. As shown in Fig. 3, the recombinant *S. avermitilis* gained the ability to produce Tü 3010 at levels comparable to those produced by *S. thiolactonus* NRRL 15439, clearly demonstrating that thiotetronate biosynthesis relies on hijacking of a sulfur supply mechanism from primary metabolism. Cosmid 8G11 was then specifically engineered, using a newly-developed system for *in vitro* CRISPR/Cas9-mediated editing (which we refer to as ICE),^52^ to reduce the insert to exactly the limits of the annotated gene cluster for Tü 3010, extending from *stuE* (asparagine synthase-like enzyme) to *stuF2* (3-oxoacyl-ACP synthase). LC-ESI-HRMS analysis of fermentation extracts of *S. avermitilis* housing truncated 8G11, which is referred to as pWHU2702 (Fig. 3) showed that this recombinant strain also produces essentially the same level Tü 3010 as does wild-type *S. thiolactonus* NRRL 15439, demonstrating that all enzymes essential for thiotetronate synthesis are likely to be encoded within this region.

### Proposed mechanism for insertion of sulfur during thiolactomycin biosynthesis invoking epoxide and thiirane intermediates

The finding that all sequenced thiotetronate gene clusters contain two cytochrome P450 genes (Fig. 2) while TLM has one is especially intriguing because TLM biosynthesis does not require oxidative steps after formation of the thiotetronate ring. Sequence comparisons of the cytochrome P450 enzymes encoded by these genes reveal that they fall into two distinct groups, as illustrated in the phylogram of Fig. S16.† In one group are TlmD1, SsuD1, StuD1, and TueD1; and in the other are SsuD2, StuD2, and TueD2. The clear implication of this is that the D2 enzymes are involved in the oxidation that leads to the carboxamide moiety in (**4**) (Fig. S17†). Meanwhile, either the D1 enzymes are inactive forms, or they are essential for a common step *prior* to the formation of the thiolactone ring and play a direct role in sulfur insertion.

**Fig. 4:**
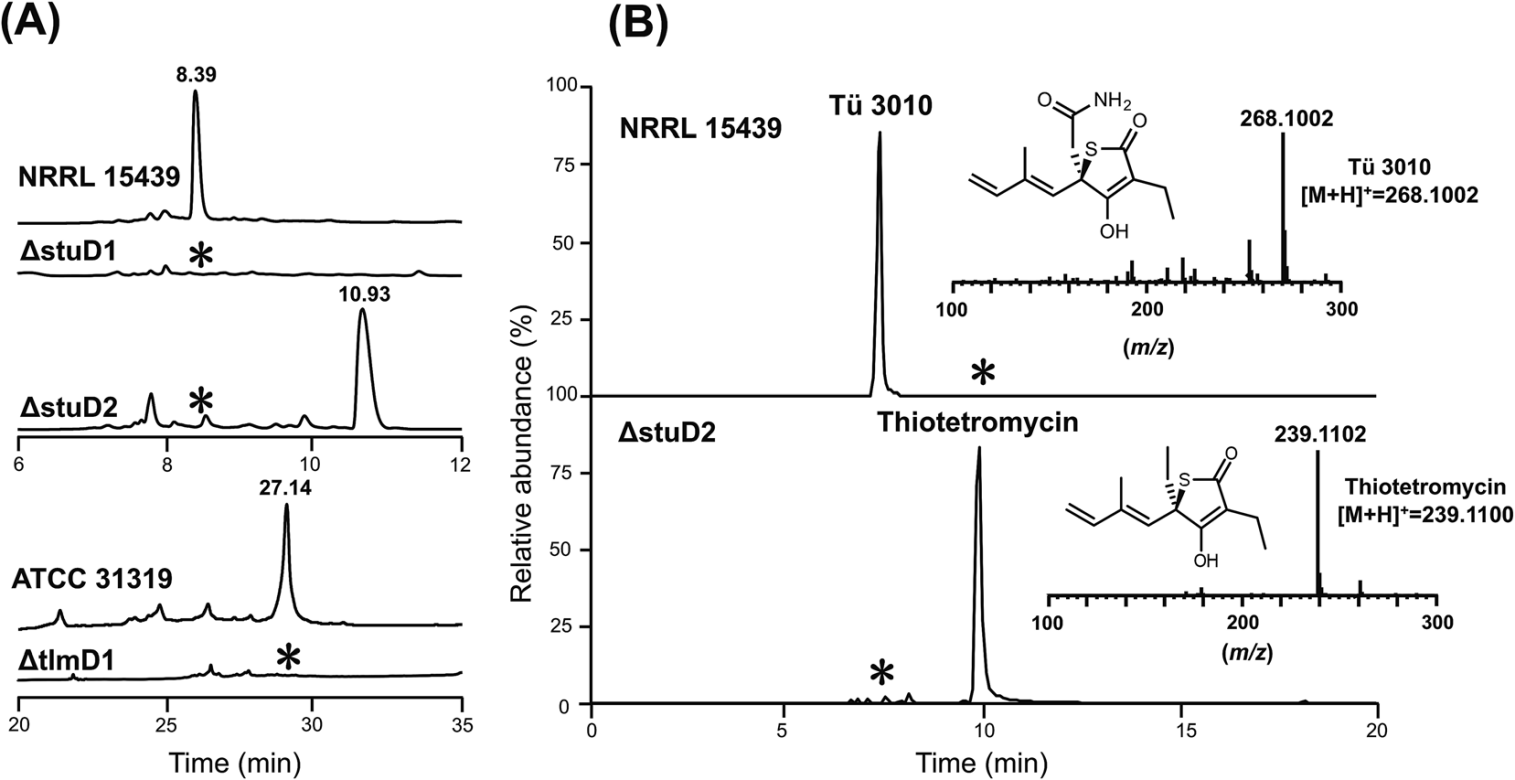
HPLC-UV, LC-ESI-HRMS and MS–MS analysis of P450 deletion mutants from both Tü 3010 and thiolactomycin (TLM) biosynthetic pathways. (A) HPLC trace profiles (UV_238 nm_) of extracts from *S. thiolactonus* wild-type strain NRRL 15439 and mutants ΔstuD1 and ΔstuD2, the *Lentzea* sp. wild-type strain ATCC31319 and ΔtlmD1 mutant. Separation was achieved as described in the materials and methods section. Production of Tü 3010 (retention time 8.39 min) was abolished in both P450 (*stuD1* and *stuD2*) mutants, although the ΔstuD2 mutant produced a new UV-absorbing peak (retention time 10.93 min). Thiolactomycin (retention time 27.14 min) production was lost, with no obvious new UV-absorbing peak, upon disruption of *tlmD1*. (B) Further LC-ESI-HRMS analysis of the ΔstuD2 intermediate by selective ion monitoring confirmed it as thiotetromycin (**2**) (Fig. S20†). Asterisk denotes not detected.

To distinguish between these possibilities, *stuD1* and *stuD2* were individually deleted in-frame, using ICE editing^52^ of the *stu* gene cluster expressed in the heterologous host strain *S. avermitilis* (Fig. S18 and S19†). LC-ESI-HRMS and MS–MS analysis of extracts from the ΔstuD2 recombinant strain showed loss of Tü 3010 production and the appearance of a new UV-absorbing peak at a retention time of 10.93 min (Fig. 4A). Further investigation of this metabolite using LC-ESI-HRMS identified its *m*/*z* as 239.1102, corresponding to the [M + H]^+^ of thiotetromycin (**2**) (Fig. 4B). Additional MS–MS and MS^3^ analysis revealed its fragmentation pattern to be analogous to that of thiolactomycin (**1**) (Fig. S20†). This provides substantial evidence that this new metabolite is the known natural thiolactone thiotetronic acid (**2**), confirming that StuD2 is involved in the oxidation of this intermediate to the carboxamide (**4**) (Fig. S20†). In contrast, LC-ESI-HRMS analysis of the ΔstuD1 mutant strain showed the abolition of Tü 3010 production but no UV-absorbance and in particular no evidence for the presence of a thiolactone (Fig. 4A).

In parallel, the single cytochrome P450 of the TLM gene cluster (*tlmD1*) was deleted in-frame and the resulting ΔtlmD1 strain was confirmed by PCR analysis (Fig. S21†). LC-ESI-MS analysis of extracts from this strain showed that thiolactomycin production was abolished (Fig. 4A). These results taken together show that both D1 and D2 enzymes are essential in their respective pathways, and that D1 acts prior to D2, and prior to formation of the thiolactone ring.

**Fig. 5:**
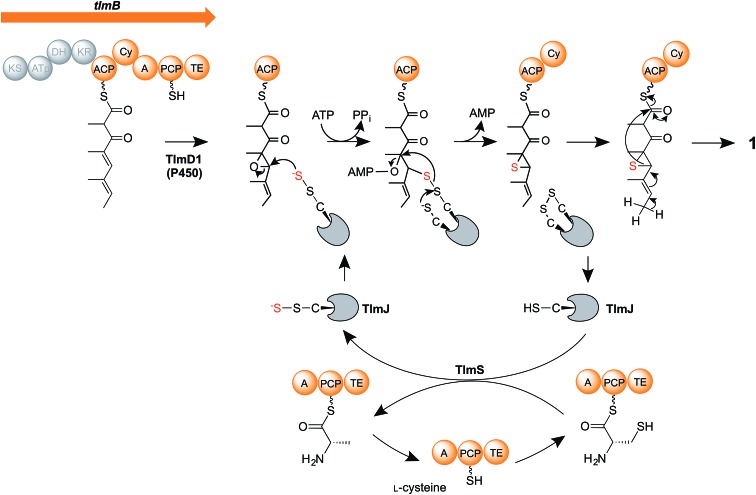
Proposed mechanism of sulfur insertion in thiolactomycin biosynthesis. For details, see the text.

We therefore propose a new mechanism (Fig. 5) for the critical steps of sulfur insertion in thiolactomycin biosynthesis that accommodates the above observations. We propose that the tetraketide formed by the action of the TLM PKS remains tethered to the ACP domain, and is selectively epoxidised by cytochrome P450 TlmD1. The chemical conversion of epoxides into thiiranes in the presence of a suitable sulfur donor such as ammonium thiocyanate proceeds in water at ambient temperature and in excellent yields.^53^ We envisage that a cysteine residue, activated by the A domain of the PKS-NRPS, becomes tethered to the PCP, and serves as the substrate for the cysteine desulfurase TlmS. The persulfide group forms at the TlmS active site, the other product being PCP-bound alanine, which we propose is hydrolysed by the adjacent thioesterase (TE) domain to allow the PCP to be re-charged with cysteine. Such hydrolytic activity is normally associated with discrete thioesterase (TEII) enzymes, which are very often found in polyketide and non-ribosomal peptide biosynthetic gene clusters and which are thought to act to remove stalled intermediates or liberate aberrantly-acylated sites.^54,55^ We propose that in addition to such an editing role in polyketide chain synthesis the discrete TE enzyme encoded in each of the Tü 3010 clusters (Fig. 2A) may similarly act to hydrolyse the alaninyl-PCP product of the cysteine desulfurase. Interestingly, the apparently redundant C-terminal TE domain in the biosynthesis of the cyanobacterial polyketide cylindrocyclophane actually resembles TEIIs and has been demonstrated to have an editing function.^56^ Meanwhile, TlmS transfers the terminal sulfur atom to TlmJ, which resembles the family of ATP-dependent thiouridylases. TlmJ as a sulfur donor catalyses nucleophilic ring-opening of the epoxide and adenylation of the hydroxyl leaving group, and subsequent ring-closure generates a thiirane, a previously-proposed intermediate.^21^ We propose that the cyclisation (Cy) domain of the PKS-NRPS then catalyses the ring-opening of the thiirane and rearrangement to form the thiolactone ring, with concomitant release of the product (**1**) from the multienzyme.

## Conclusion

Polyketide natural products containing sulfur include a number of interesting and valuable chemotypes, and prominent among these are the anticancer prodrug leinamycin,^57^ the thiosugar-containing antibiotic BE-7585,^51^ and the thiotetronates. In many cases the enzymatic machinery is shared with or borrowed from primary metabolic pathways for sulfur delivery.^48,51^ In the case of leinamycin, however, it has been recently shown that a cysteine desulfurase domain integrated into the assembly-line multienzyme is responsible for sulfur insertion.^58^ The comparative genomics approach used here has provided the first detailed insights into the novel enzymatic machinery for assembly of thiotetronates, including the nature of the sulfur donors. The PKS-NRPS multienzyme contains only one PKS extension module rather than the three expected from the chemical structure, partly explaining why thiotetronate gene clusters have eluded detection until now. The multienzyme also features domains predicted to tether cysteine, an arrangement perhaps serving to sequester this sulfur donor and channel sulfur more effectively towards thiotetronate biosynthesis. We propose a mechanism for sulfur insertion and thiolactone formation involving an epoxide intermediate. Efforts to detect this and other enzyme-bound intermediates are in progress. The stage is also now set for reconstitution of these pathways *in vitro*, investigation of the structure and mechanism of key enzymes, and manipulation of the genes *in vivo* to produce analogues of potential therapeutic value.

## Materials and methods

### Fermentation, isolation, and identification of the thiotetronates

For analysis of thiolactomycin and Tü 3010 production, strains were cultured in liquid production medium for 4 days. The fermentation broth was adjusted to pH 3, shaken vigorously with an equal volume of ethyl acetate for 2 hours (37 °C, 250 rpm), then clarified by centrifugation. The resulting supernatant was then evaporated to dryness under reduced pressure in a rotary evaporator, the crude extract was dissolved in 600 μl of methanol, and clarified by centrifugation (12 000 × *g*, 10 min).

LC-ESI-HRMS analysis of Tü 3010 was performed on LTQ XL Orbitrap (Thermo Scientific) coupled to a Thermo Instruments HPLC system, operated in positive ion mode with electrospray ionization. HPLC separation was achieved using a Phenomenex C18 column (4.6 × 250 mm, 5 μm) equilibrated with 20 mM ammonium acetate (A) and methanol (B), and was developed with the following program: 0–2 min, 90% A; 2–4 min, 90% A – 40% A; 4–15 min, 40% A – 20% A; 15–16 min, 20% A – 10% A; 16–20 min, 10% A – 90% A. The flow rate was 1 mL min^–1^ and UV absorption was monitored at 238 nm and 303 nm.

LC-ESI-MS analysis of thiolactomycin was performed on a Finnigan LTQ mass spectrometer (Thermo Scientific), operating in positive ion mode. It was coupled to a HP1100 HPLC system (Agilent), fitted with a Phenomenex Prodigy C18 column (4.6 × 250 mm, 5 μm). Separation was achieved using a solvent system of acetonitrile and H_2_O (both containing 0.1% formic acid) with a linear gradient of 3–100% of acetonitrile over 42 min at a flow rate of 0.7 mL min^–1^.

### *In vitro* engineering of the core *stu* gene cluster

The Type II Clustered Regularly Interspaced Short Palindromic Repeats (CRISPR)/CRISPR-associated proteins (Cas) of *Streptococcus pyogenes* constitute a type of bacterial immune response, targeting cleavage of DNA perceived as foreign. RNA-guided Cas9 cleavage has been developed as the basis for highly precise and convenient genome engineering.^59–61^ In this work an *in vitr*o CRISPR/Cas9-mediated editing (ICE) procedure^52^ was used to specifically reduce the size of the DNA insert on 8G11 to the limits of the annotated *stu* cluster (*stuE-stuF2*). Cosmid 8G11 was first treated with Cas9, guided by gRNA-stuF2O-1 and gRNA-stuF2O-2 to generate a linear DNA fragment of interest lacking the right unwanted arm (∼10 kbp) of the insert. After end-repair using T4 DNA polymerase, the resulting linear DNA was then cyclised. The cyclised recombinant was again treated with Cas9, guided by gRNA-stuEO-1 and gRNA-stuEO-2, to create the linear DNA fragment now also lacking the left unwanted arm (∼5 kbp) of the insert. After end-repair, cyclisation generated pWHU2702, housing the annotated set of *stu* genes only.

To study the role of the *stuD1* and *stuD2* cytochrome P450 genes in Tü 3010 biosynthesis, in-frame deletion recombinants for individual genes in the *stu* cluster were constructed using the ICE system described above. To construct pWHU2712 (ΔstuD1) for example, pWHU2702 was treated with Cas9, guided by gRNA-stuD1-1 and gRNA-stuD1-2, and the resulting linear fragment was end-repaired and self-ligated to create pWHU2712. The same procedure was used with guide oligonucleotides gRNA-stuD2-1 and gRNA-stuD2-2 to generate a plasmid-borne *stu* cluster housing ΔstuD2. The in-frame deletion recombinant plasmids were confirmed by restriction digestion and sequencing of the editing joins. The recombinant plasmids were then each introduced into *S. avermitilis* MA-4680 by conjugation, and the resulting recombinant strains were analysed for the heterologous production of Tü 3010.

### Sequencing and bioinformatic analysis

Whole genome sequencing for *S*. *olivaceus* Tü 3010 and *Lentzea* sp. ATCC 31319 was carried out by the DNA Sequencing Facility in the Department of Biochemistry, University of Cambridge, and whole genome sequencing for *S*. *thiolactonus* NRRL 15439 and *Streptomyces* sp. MG11 was commercially performed by Shanghai Southgene Technology Co., Ltd. (SSGT). DNA was analysed and annotated using Artemis Release 13.0.^28^ The bioinformatics program antiSMASH was used in initial identification and analysis of genetic clusters.^3,62^ Functional analysis of each gene product was carried out using BlastP^30^ and multiple protein alignments were compiled by Clustal Omega.^63^ Predictions for PKS and NRPS domains were made using the University of Maryland PKS/NRPS Analysis Website.^64^ The structures of molecules were drawn and chemical compound masses calculated using ChemBioDraw Ultra version 14.0 (Perkin-Elmer Informatics). The biosynthetic gene clusters (bio), as well as each corresponding sulfur cluster (sulfur), of the thiotetronate antibiotics have been deposited in the EMBL database with the following accession numbers: *S*. *olivaceus* Tü 3010, LN879414 (bio) and LN879415 (sulfur); *Lentzea* sp. ATCC 31319, LN879412 (bio) and LN879413 (sulfur); *S*. *thiolactonus* NRRL 15439, LN879418 (bio) and LN879419 (sulfur); *Streptomyces* sp. MG11, LN879416 (bio) and LN879417 (sulfur).
