# Supplementary material for: A genomics-led approach to deciphering the mechanism of thiotetronate antibiotic biosynthesis
Source: Chem Sci. 2015 Oct 8;7(1):376–85. doi: 10.1039/c5sc03059e (PMC5518548; doi:10.1039/c5sc03059e)
Supplement: Supplementary file 1 [file SC-007-C5SC03059E-s001.pdf]

Electronic Supplementary Material (ESI) for Chemical Science.
This journal is © The Royal Society of Chemistry 2015

Supporting Information

A genomics-led approach to deciphering the mechanism of thiotetronate antibiotic biosynthesis

Weixin Tao,‡[a] Marie E. Yurkovich,‡[b] Shishi Wen,‡[a] Karen E. Lebe,[b] Markiyan Samborskyy,[b] Yuanzhen Liu,[a] Anna Yang,[a] Yunkun Liu,[a] Yingchen Ju,[a] Zixin Deng,[a] Manuela Tosin,[c] Yuhui Sun[a] and Peter F. Leadlay[b]

[a] *Key Laboratory of Combinatorial Biosynthesis and Drug Discovery (Wuhan University), Ministry of Education, and Wuhan University School of Pharmaceutical Sciences, Wuhan 430071, People's Republic of China*

[b] *Department of Biochemistry, University of Cambridge, Sanger Building, 80 Tennis Court Road, Cambridge CB2 1GA, United Kingdom*

[c] *Department of Chemistry, University of Warwick, Library Road, Coventry CV4 7AL, United Kingdom*

‡ These authors contributed equally to this work

Correspondence:

*Professor Yuhui Sun: E-mail: yhsun@whu.edu.cn

*Professor Peter F. Leadlay: E-mail: pfl10@cam.ac.uk

Contents of Supporting Information

Experimental Procedures..4

Supplementary Results..8

Table S1. ^{1}H and ^{13}C NMR data of compound 4 from *Streptomyces thiolactonus* NRRL 15439 (600 MHz) and *Streptomyces* sp. MG11 (400 MHz)..8

Table S2. Comparison of the thiotetronate gene clusters from four different producer strains..9

Table S3. Bacterial strains used in this study..10

Table S4. Plasmids used in this study..12

Table S5. Oligonucleotide primers used in this study..............................13

Figure S1. Selected NMR spectra for compound 4 from *Streptomyces thiolactonus* NRRL 15439..16

Figure S2. Selected NMR spectra for compound 4 from *Streptomyces* sp. MG11..19

Figure S3. Chemical structures and key ^{1}H-^{1}H COSY and HMBC correlations of compound 4 from *Streptomyces thiolactonus* NRRL 15439 and *Streptomyces* sp. MG11..22

Figure S4. Sequence alignment of acyltransferase (AT) domains from thiotetronate PKS enzymes..23

Figure S5. Biosynthesis of the Tü 3010 tetraketide backbone by the iterative PKS TueB..24

Figure S6. Sequence alignment of FabB/FabF enzymes from thiotetronate gene clusters..25

Figure S7. Sequence alignment of FabH enzymes from thiotetronate gene clusters..26

Figure S8. In-frame deletion of *stuH* in *S. thiolactonus*...........................27

Figure S9. In-frame deletion of *stuB* in *S. thiolactonus*...........................28

Figure S10. In-frame deletion of *tlmA* in *Lentzea* sp. 29
Figure S11. LC-ESI-MS analysis of thiolactomycin (TLM) from *Lentzea* sp...30
Figure S12. Genetic analysis of genes of sulfur metabolism involved in Tü 3010 biosynthesis..31
Figure S13. Replacement of *stuJ,K,S* genes by chloramphenicol resistance gene (*cat*) in *S. thiolactonus*..32
Figure S14. In-frame deletion of *stuK* in *S. thiolactonus*..........................33
Figure S15. In-frame deletion of *stuS* in *S. thiolactonus*..........................34
Figure S16. Phylogram of cytochrome P450 enzymes encoded in thiotetronate biosynthetic gene clusters 35
Figure S17. Proposed mechanism for formation of the carboxamide function of Tü 3010..36
Figure S18. In-frame deletion of the cytochrome P450-encoding gene *stuD2* in pWHU2702...37
Figure S19. In-frame deletion of the cytochrome P450-encoding gene *stuD1* in pWHU2702...38
Figure S20. Fragmentation pathway for (A) thiotetromycin 39
Figure S21. In-frame deletion of *tlmD1* in *Lentzea* sp. ATCC3139…........40
References...41

Materials and Methods

Bacterial Strains and Culture Conditions. Bacterial strains and plasmids used in this study are summarised in Tables S2 and S3 respectively. *S. olivaceus* Tü 3010, *Lentzea* sp. ATCC 31319, *S. thiolactonus* NRRL 15439, *Streptomyces* sp. MG11 and related mutant strains were grown in TSBY or TSB medium[1] for isolation of chromosomal DNA, and on SFM medium[1] for sporulation and conjugation. For production of Tü 3010 in *S. olivaceus* Tü 3010 the strain was cultured in 2% oatmeal complex medium with a trace element solution,[2] for production of TLM, *Lentzea* sp. ATCC 31319 was cultured in TSB, and for production of Tü 3010 in *S. thiolactonus* NRRL 15439 and *Streptomyces* sp. MG11 strains were cultured in ISP2.[1] For liquid cultures, the strains were grown at 30°C on a rotary shaker incubator at 200 rpm for 4 days, and for solid culture, the strains were grown at 30°C for 5-7 days. All *E. coli* strains were grown in liquid or solid Luria-Bertani (LB) medium at 37°C with appropriate antibiotic selection (apramycin 50 µg/mL, chloramphenicol 25 µg/mL, kanamycin 50 µg/mL).

DNA Manipulation. All chemicals and antibiotics were purchased from Sigma-Aldrich and all organic solvents were HPLC grade. Fast-digest restriction endonucleases were purchased from Thermo Fisher Scientific. Alkaline phosphatase, T4 DNA ligase, Gibson Assembly Master Mix, T4 DNA polymerase, Deoxynucleotide (dNTP) Solution Mix, and bovine serum albumin (BSA) were purchased from New England Biolabs. Oligonucleotides used in this work were custom synthesized by Eurofins Genomics, GenScript, or Tsingke and supplied in lyophilised form (listed in Table S4). Polymerase chain reactions were carried out using *Phusion* High-Fidelity PCR Master Mix from New England Biolabs (for cloning), BioMix Red from Bioline and Taq PCR Master Mix from YPH-Bio (for screening). Isolation of plasmid DNA from an overnight culture was performed using the E.Z.N.A. HP Plasmid Mini Kit I

(Omega Bio-Tek). High-molecular weight genomic DNA from *Streptomyces* and *Lentzea* strains was isolated using the salting out procedure.[1] Purification of DNA fragments from agarose gels was performed using GeneJet Gel Extraction Kit (Thermo Fisher Scientific) according to the manufacture's instruction.

Genomic Library Construction and Library Screening for *stu* Gene Cluster. A genomic library of *S. thiolactonus* NRRL 15439 was constructed using CopyControl™ Fosmid Library Production Kit (EPICENTRE Biotechnologies) according to the manufacturer's instructions. High-molecular weight genomic DNA of *S. thiolactonus* NRRL 15439 was sheared to ~40 kbp by passing through a syringe needle for 10-15 times. DNA fragments range from 35 kbp to 45 kbp were size-fractionated by low-melting agarose gel electrophoresis and recovered by ethanol precipitation after agarase (Thermo Fisher Scientific) digestion. End-repair of insert DNA was performed using End-repair Enzyme Mix supplied in Fosmid Library Production Kit. End-repaired DNA was then ligated with EcoRV linearized and FastAP dephosphorylated vector pJTU2554.[3] The ligation mixture was packaged using MaxPlax Lambda Packaging Extracts and plated with *E. coli* EPI300/pUZ8002. Screening of the *S. thiolactonus* genomic library for the *stu* cluster was performed by PCR using primer pairs stuE-up/stuE-dn, stuH-up/stuH-dn, stuA-up/stuA-dn, and stuC-up/stuC-dn.

Genetic manipulation of thiotetronate-producing strains.

For *Lentzea* sp. ATCC 31319, in-frame deletions of the *tlmA* and *tlmD1* genes were carried out. The recombinant plasmids pMYΔtlmA and pMYΔtlmD1 were constructed by amplifying regions upstream and downstream of *tlmA* (1.5 kbp on each) and *tlmD1* (2 kbp on each) using *Lentzea* sp. genomic DNA and the primer pairs: tlmA-1F/tlmA-1R, tlmA-2F/tlmA-2R, tlmD1-1F/tlmD1-1R, tlmD1-2F/tlmD1-2R. The vector pYH7[4] was digested with BglII and HindIII,

treated with alkaline phosphatase, and purified by gel electrophoresis. Ligation of cut pYH7 and the amplified fragments was accomplished by the isothermal Gibson assembly method according to the manufacturer's protocol. The assembly mixture was incubated at 50°C for 60 min, and then was used to transform *E. coli* DH10B. Plasmids pMYΔtlmA and pMYΔtlmD1 were confirmed by sequencing and were each introduced by conjugation into *Lentzea* sp. ATCC 31319 through donor strain *E. coli* ET12567/pUZ8002. After incubation at 30°C for 16 hours, exconjugants were selected with 5 μg/mL apramycin and 25 μg/mL nalidixic acid. Antibiotic resistance was confirmed through transfer of exconjugants to a SFM plate containing 50 μg/mL apramycin and 25 μg/mL nalidixic acid. To form the double cross-over mutants, loss of pMYΔtlmA and pMYΔtlmD1 was obtained by several rounds of non-selective growth of the exconconjugants on SFM agar medium. The integrity of the *Lentzea* sp. ΔtlmA and ΔtlmD1 mutants were checked by PCR analysis using the primer pairs: tlmA-3F/tlmA-3R and tlmD1-3F/tlmD1-3R.

For *S. thiolactonus* NRRL 15439, gene replacement of *stuJ,K,S*, and four respective gene in-frame deletions of *stuK, stuS, stuH, stuB* were carried out. Taking replacement of *stuJ,K,S* as an example, the recombinant plasmid pWHU2617 was constructed by amplifying 2 kbp regions upstream and downstream of the *stuJ,K,S* gene, using *S. thiolactonus* genomic DNA and the primer pairs: stuJ,K,S-L1/stuJ,K,S-L2 and stuJ,K,S-R1/stuJ,K,S-R2. The pYH7[4] fragment was prepared by digesting with BglII and NdeI, and subsequently treating with alkaline phosphatase. Amplified fragments (2 kbp) after digestion were cloned into BglII and NdeI sites of pYH7 to give an intermediate recombinant. A 827 bp *cat* resistance gene cassette was amplified using primer pairs: cat-up/cat-dn. Plasmid pWHU2617 was created by inserting the *cat* cassette between the two 2 kbp amplified fragments. Restriction digestion and sequencing of inserts were performed to confirm the recombinant plasmid pWHU2617, which was then introduced into *S. thiolactonus* NRRL 15439 by conjugation. Screening for gene replacement

mutant of *stuJ,K,S* was performed as described for ΔtlmA mutant screening. Recombinant strain *S. thiolactonus* ΔstuJ,K,S::*cat* was confirmed by PCR using primer pairs: stuJ,K,S-CK1/stuJ,K,S-CK2 and Southern blot analysis. The in-frame deletion of each of *stuK, stuS, stuH, stuB* were carried out similarly. Recombinant plasmids, pWHU2639, pWHU2640, pWHU2674 and pWHU2673 were constructed by amplifying 2 kbp regions upstream and downstream of the target gene, using the primer pairs: stuK-L1/stuK-L2 and stuK-R1/stuK-R2 (*stuK*), stuS-L1/stuS-L2 and stuS-R1/stuS-R2 (*stuS*), stuH-L1/stuH-L2 and stuH-R1/stuH-R2 (*stuH*), stuB-L1/stuB-L2 and stuB-R1/stuB-R2 (*stuB*), respectively. The resulting recombinant plasmids were each introduced into *S. thiolactonus* and screened for the in-frame deletion mutants of interest, as described previously. All mutants were validated by PCR analysis, using the check primers listed in Table S4.

Trans-complementation was carried out by introducing the complementation plasmids pIB-tlmA, pWHU2698, pWHU2699, pWHU2700 and pWHU2701 into the corresponding gene-null mutant. The integrative vector pIB139 was used for inserting *tlmA, stuJ*, *stuK*, *stuS*, *stuJ+S* under the control of the *ermE** promoter, respectively. Genes including *tlmA* (2 kbp), *stuJ* (1 kbp), *stuK* (666 bp) and *stuS* (1 kbp), were amplified using primer pairs: tlmA-NdeI/tlmA-XbaI, stuJ-up/stuJ-dn, stuK-up/stuK-dn, stuS-up/stuS-dn, respectively. The resulting products were digested with NdeI and XbaI, and then cloned into the corresponding site of pIB139, except for *stuS* which was digested with NdeI and EcoRI and inserted into the NdeI and EcoRI site of pIB139. After sequencing confirmation, each plasmid was introduced into the appropriate gene-null mutant and analyzed for the production of thiotetronate antibiotic.

Table S1. ^{1}H and ^{13}C NMR data of compound 4 from *Streptomyces thiolactonus* NRRL 15439 (600 MHz) and *Streptomyces* sp. MG11 (400 MHz).

position	Tü 3010 (from MG11)		Tü 3010 (from NRRL 15439)	
	δ_H (*mult*, *J* in Hz)	δ_C (type)	δ_H (*mult*, *J* in Hz)	δ_C (type)
1	/	195.3, C	/	195.2, C
2	/	114.7, C	/	115.0, C
3	/	183.0, C	/	181.7, C
4	/	55.4, C	/	55.2, C
5	5.73 (s)	129.0, CH	5.72 (s)	128.8, CH
6	/	138.9, C	/	139.0, C
7	6.37 (dd, 17.2,10.5)	140.8, CH	6.36 (dd, 17.3,10.6)	140.8, CH
8	5.27 (d, 17.2);<br>5.05 (d, 10.5);	112.3, CH_2	5.26 (d, 17.3);<br>5.04 (d, 10.6);	112.4, CH_2
9	1.77 (s, 3H)	11.1, CH_3	1.76 (s, 3H)	11.1, CH_3
10	3.20 (d, 16.0);<br>3.06 (d, 16.0);	45.3, CH_2	3.22 (d, 16.1);<br>3.06 (d, 16.1);	45.2, CH_2
11	/	174.1, C	/	174.2, C
12	2.19 (dq, 15.0, 6.7, 2H);	15.7, CH_2	2.21 (m, 2H);	15.7, CH_2
13	1.02 (t, 6.7, 3H)	11.3, CH_3	1.01 (t, 7.5, 3H)	11.2, CH_3

* Recorded in CD_3OD, 25℃, δ in ppm

Table S2. Comparison of the thiotetronate gene clusters from four different producing strains.

***S. thiolactonus* NRRL 15439**		***Streptomyces* sp. MG11**		***S. olivaceus* Tü 3010**		***Lentzea* sp. ATCC 31319**		**Putative function**
gene	No. (aa)	gene	No. (aa)	gene	No. (aa)	gene	No. (aa)	
stuJ	376	*ssuJ*	376	*tueJ*	367	*tlmJ*	335	tRNA-specific 2-thiouridylase
stuK	221	*ssuK*	222	*tueK*	227	/		*N*-acetylmuramoyl-L-alanine amidase
stuS	389	*ssuS*	391	*tueS*	390	*tlmS*	379	NifS-like cysteine desulfurase
stuE	611	*ssuE*	611	*tueE*	611	/		Asparagine synthase
stuG	62	*ssuG*	62	*tueG*	62	/		Ferredoxin
stuD2	403	*ssuD2*	405	*tueD2*	406	/		Cytochrome P450
stuF3	337	*ssuF3*	325	/		/		3-oxoacyl-ACP synthase (FabH)
stuT	259	*ssuT*	252	*tueT*	252	/		Thioesterase
stuH	444	*ssuH*	444	*tueH*	447	/		Carboxylating enoyl-CoA reductase (Ccr)
stuI	583	*ssuI*	586	*tueI*	581	/		3-hydroxybutyryl-CoA dehydrogenase
stuR	807	*ssuR*	850	*tueR*	801	/		LuxR family transcriptional regulator
stuF1	401	*ssuF1*	401	*tueF*	390	*tlmF*	407	3-oxoacyl-ACP synthase (FabF/FabB)
stuD1	424	*ssuD1*	424	*tueD1*	424	*tlmD1*	384	Cytochrome P450
stuA	698	*ssuA*	690	*tueA*	689	*tlmA*	647	Polyketide synthase (PKS)
stuB	2286	*ssuB*	2265	*tueB*	2274	*tlmB*	2972	PKS/NRPS
stuC	634	*ssuC*	894	*tueC*	632			Nonribosomal peptide synthetase (NRPS)
stuF2	369	*ssuF2*	404	/		/		3-oxoacyl-ACP synthase (FabF/FabB)

"/" indicates not present

Table S3. Bacterial strains used in this study.

Strains	Characteristics	Reference
Escherichia coli		
DH10B	F^- *mcrA* Δ(*mrr*$^-$ *hsd* RMS$^-$ *mcrBC*), Φ80*lacZ* ΔM15, Δ*lacX74 recA1* endA1 *araD139* Δ (*ara leu*)7697 *galU* galK *rpsL nupG* λ^-, Host for general DNA manipulation	Invitrogen
EPI300	Host for constructing the genomic library	Epicentre Biotechnologies
ET12567/pUZ8002	(F^- *dam*$^-$ 13::Tn9 *dcm*$^-$ 6 *hsdM hsdR recF143* zjj-202::Tn10 *galK2 galT22 ara14 pacY1 xyl-5 leuB6 thi-1*), Donor strain for conjugation between *E. coli* and *Streptomyces.*	[5]
***Lentzea* sp.**		
Lentzea sp. ATCC 31319	TLM producing wild-type strain	[6]
ΔtlmA	*tlmA* gene in-frame deletion mutant	This study
ΔtlmD1	*tlmD1* gene in-frame deletion mutant	This study
ΔtlmA::pIB-tlmA	*tlmA* gene in-frame deletion mutant complemented with *tlmA* gene	This study
Streptomyces		
S. olivaceus Tü 3010	Tü 3010 producing wild-type strain	[2]
S. thiolactonus NRRL 15439	Tü 3010 producing wild-type strain	[7]
Streptomyces sp. MG11	Tü 3010 producing wild-type strain	This study
ΔstuJ,K,S	*stuJ,K,S* genes were replaced by *cat*	This study
ΔstuK	*stuK* gene in-frame deletion mutant	This study
ΔstuS	*stuS* gene in-frame deletion mutant	This study
ΔstuH	*stuH* gene in-frame deletion mutant	This study
ΔstuB	*stuB* gene in-frame deletion mutant	This study
ΔstuJ,K,S::pWHU2698	*stuJ,K,S* genes replacement mutant complemented with *stuJ* gene	This study
ΔstuJ,K,S::pWHU2699	*stuJ,K,S* genes replacement mutant complemented with *stuK* gene	This study
ΔstuJ,K,S::pWHU2700	*stuJ,K,S* genes replacement mutant complemented with *stuS* gene	This study
ΔstuJ,K,S::pWHU2701	*stuJ,K,S* genes replacement mutant complemented with *stuJ* and *stuS*	This study

S. avermitilis		
S. avermitilis MA-4680	Heterologous host for Tü 3010	8
MA-4680::pJTU2554	*S. avermitilis* housing pJTU2554	This study
MA-4680::8G11	*S. avermitilis* housing cosmid 8G11	This study
MA-4680::19H12	*S. avermitilis* housing cosmid 19H12	This study
MA-4680::pWHU2702	*S. avermitilis* housing the pWHU2702, containing the core *stu* cluster	This study
MA-4680::pWHU2705	*S. avermitilis* housing the core *stu* cluster with the *stuD2* gene deleted in-frame	This study
MA-4680::pWHU2712	*S. avermitilis* housing the core *stu* cluster with the *stuD1* gene deleted in-frame	This study

Table S4. Plasmids used in this study.

Plasmid	Characteristics	Reference
pJTU2554	Vector for construction of genomic library	[3]
pUC18	Vector for general cloning	[9]
pYH7	*E. coli-Streptomyces* shuttle vector, for in-frame gene deletions	[4]
pIB139	*ΦC31* site integrative vector with P*ermE** promoter, for *in trans* gene complementation	[10]
pMYΔtlmA	Construct for in-frame deletion of *tlmA*	This study
pMYΔtlmD1	Construct for in-frame deletion of *tlmD1*	This study
pIB-tlmA	Construct with *tlmA* under control of PermE*, for ΔtlmA complementation	This study
8G11	*S. thiolactonus* NRRL 15439 library cosmid with *stu* cluster	This study
19H12	*S. thiolactonus* NRRL 15439 library cosmid with *stu* cluster	This study
pWHU2617	Construct for in-frame deletion of *stuJ,K,S*	This study
pWHU2639	Construct for in-frame deletion of *stuK*	This study
pWHU2640	Construct for in-frame deletion of *stuS*	This study
pWHU2698	Construct with *stuJ* under control of the PermE*, for ΔstuJ,K,S complementation	This study
pWHU2699	Construct with *stuK* under control of the PermE*, for ΔstuJ,K,S complementation	This study
pWHU2700	Construct with *stuS* under control of the PermE*, for ΔstuJ,K,S complementation	This study
pWHU2701	Construct with *stuJ* and *stuS* under control of the PermE*, for ΔstuJ,K,S complementation	This study
pWHU2673	Construct for in-frame deletion of *stuB*	This study
pWHU2674	Construct for in-frame deletion of *stuH*	This study
pWHU2702	Construct housing the core *stu* cluster (*stuE* to *stuF2*) for Tü 3010 heterologous expression	This study
pWHU2705	Construct housing the core *stu* cluster with *stuD2* in-frame deleted	This study
pWHU2712	Construct housing the core *stu* cluster with *stuD1* in-frame deleted	This study

Table S5. Oligonucleotide primers used in this study.

Primer	oligonucleotide sequence(5’-3’)	Restriction site
tlmA-1F	GTGCCTCCCCACTCCTGCAGATCTAAATAGGATTTCTGCTGTTCGACCGGAACGTCC	
tlmA-1R	GTCCACCAGTTCGAAGAAGCCCGAGTCGAAGGCGTC	
tlmA-2F	TCGGGCTTCTTCGAACTGGTGGACCGCATCAACACC	
tlmA-2R	GTCGACCTGCAGGCATGCAAGCTTTGGTCTTCGCCGACAGCGTCAGC	
tlmD1-1F	GTGCCTCCCCACTCCTGCAGATCTAAGCAAAGCGCAGCGTGAAC	
tlmD1-1R	CAACGCCTCGAAGCAGATCACCTCGATCGAGAACG	
tlmD1-2F	GAGGTGATCTGCTTCGAGGCGTTGATCGAACG	
tlmD1-2R	GTCGACCTGCAGGCATGCAAGCTTTGTTGATGCGGTCCACCAGTTCC	
stuJ,K,S-L1	GTCCATATGGGCGGCGGAGAGGAAGAC	NdeI
stuJ,K,S-L2	GATAGATCTGACCAGGCGAGCGACGGC	BglII
stuJ,K,S-R1	GGTAGATCTGATTCTACGGCTCCCCTC	BglII
stuJ,K,S-R2	ATGAAGCTTCTGCCCGCCCCGCACCGC	HindIII
stuJ-L1	CCCAAGCTTAGGGGCGAGTGCGAGAAGAC	HindIII
stuJ-L2	CCGGAATTCGCACCTACACGGCCCAGCTC	BamHI
stuJ-R1	CCGGAATTCAGAAGGGGATGCCGATGACG	BamHI
stuJ-R2	CGCGGATCCCGTCCTCCCTCCACGCATCC	EcoRI
stuK-L1	CCCAAGCTTCGGCACCCGCATCGCCTACG	HindIII
stuK-L2	CCGGAATTCTCCGTGTCCTTGGATGCTCG	EcoRI
stuK-R1	CCGGAATTCAGGACTTCACGGACGAGATG	EcoRI
stuK-R2	CGCGGATCCAATCGTTCACCGAAACCCTG	BamHI
stuS-L1	CCCAAGCTTATCGTCGCCGAGCCCACCAC	HindIII
stuS-L2	CGCCATATGCCGGCCAACGCGCATTTCAC	NdeI
stuS-R1	CGCCATATGGCGGCGTGACCAGTAAAGGC	NdeI
stuS-R2	CGCGGATCCCGAACTCGCCGACGCCTGGG	BamHI
stuB-L1	CCCAAGCTTGCCGCGCGGGACTCCTGTAC	HindIII
stuB-L2	CCGGAATTCCCGCACCCCGCAAGTCATCC	EcoRI
stuB-R1	CCGGAATTCCTGACGATCATGATGGCCTC	EcoRI
stuB-R2	CGCCATATGAGCCTTCAAACCCTACCTCG	NdeI
stuH-L1	CCCAAGCTTTGTCCGTCGCCACGCTGCCC	HindIII
stuH-L2	CCGGAATTCCCGGGTGCCAGGCGTTGACG	EcoRI

stuH-R1	CCGGAATTCGCAACCTCCACCAGGGCAAG	EcoRI
stuH-R2	CGCCATATGCGGGAGGGTCTCACCAATCG	NdeI
tlmA-3F	AGTTCCTGCGCAACCTCGTCG	
tlmA-3R	AGAGATGTTCCGGTGTGTCG	
tlmD1-3F	ATGGTGTACGCCGCCATCAGGAAGG	
tlmD1-3R	AGATCGCGCTCATCGGGTCACCTCG	
stuJ,K,S-CK1	GTGCTGGTGGAGTTCTTC	
stuJ,K,S-CK2	GCGTCCTTCATCCTGGGC	
stuJ-CK1	GAAGGTCCGCAGCGTCAGGC	
stuJ-CK2	CCCTCCTTGCGCGGCTTCTC	
stuK-CK1	CGCCCGTGCGGAAGGACTGG	
stuK-CK2	CGCGCATTTCACGTTCCCCG	
stuS-CK1	GCCGAGGGAGAAGCGGAGGG	
stuS-CK2	GCTGCTGGGGCTCGGTCTCG	
stuB-CK1	CGAAACGGCGATGGAGGACG	
stuB-CK2	GACATCGCCGTCCCCTTCGC	
stuH-CK1	CACGCAAGTCCCTTCACCTC	
stuH-CK2	AGGGAAGAGAGGCAGCGAGG	
tlmA-NdeI	AACATATGATGAGCGCGATCGCCGTGATC	NdeI
tlmA-XbaI	AATCTAGATCAGGCACAGCTCGCCTCCATC	XbaI
cat-up	GGAAGATCTAGGTTCCAACTTTCAC	BglII
cat-dn	GGAAGATCTCAAAATCATACCTGACC	BglII
stuJ-up	CGCCATATGATGACTCACACCCCGCAG	NdeI
stuJ-dn	CCCAAGCTTTCAGGCCACGCCCGCCGT	EcoRI
stuK-up	CGCCATATGATGGGGGCGAGACGAGCA	NdeI
stuK-dn	CCCAAGCTTTCAGGCCGGCGACGTACG	EcoRI
stuS-up	CCGGAATTCCTACGTCAGCCCCGCCGC	NdeI
stuS-dn	CGCCATATGATGGCTTACCTCGACCAC	EcoRI
stuA-up	CGCCATATGATGACCGCACTGACACACCA	NdeI
stuA-dn	CCCAAGCTTTCAGCCACGGAGTGCCTCCA	HindIII
stuC-up	CGCCATATGGTGAACTCTGTCGCTAACTC	NdeI
stuC-dn	CCCAAGCTTTCACGCGGCCGCGCGGGACT	HindIII
stuH-up	CGCCATATGGTGAAGGAAATAGTCGATGC	NdeI
stuH-dn	CCCAAGCTTTCACGGCCGGAAGCGGTTGA	HindIII
stuE-up	CGCCATATGATGACCGAGACCATGGCCTG	NdeI
stuE-dn	CCCAAGCTTTCAGTTGAGCTTCAGCGTCG	HindIII
gRNA18-1/A	AAAAGCACCGACTCGGTGCCACTTTTTCAAG<br>TTGATAACGGACTAGCCTTATTTTAACTTGCT	
gRNA-18-1/S	GATCACTAATACGACTCACTATA**GGCCAACGC**<br>**GCGGGGAGAGG**GTTTTAGAGCTAGAAATAGC<br>AAGTTAA	

gRNA-stuD1-1/S	GATCACTAATACGACTCACTATA**GGGCCGGAACGCAGCGGTC**GTTTTAG
gRNA-stuD1-2/S	GATCACTAATACGACTCACTATA**GCGCAAGTTCGGCGGCAGGT**GTTTTAG
gRNA-stuD2-1/S	GATCACTAATACGACTCACTATA**GAGAAAGGACAGCCCGGCGG**GTTTTAG
gRNA-stuD2-2/S	GATCACTAATACGACTCACTATA**GCCATGAACTCCCGGTCACC**GTTTTAG

Note: Restriction sites introduced into primers are underlined; guide sequences in gRNA primers are shown in bold type.

Fig. S1. Selected NMR spectra for compound 4 from *Streptomyces thiolactonus* NRRL 15439.

a) ^{1}H-NMR spectrum of compound 4

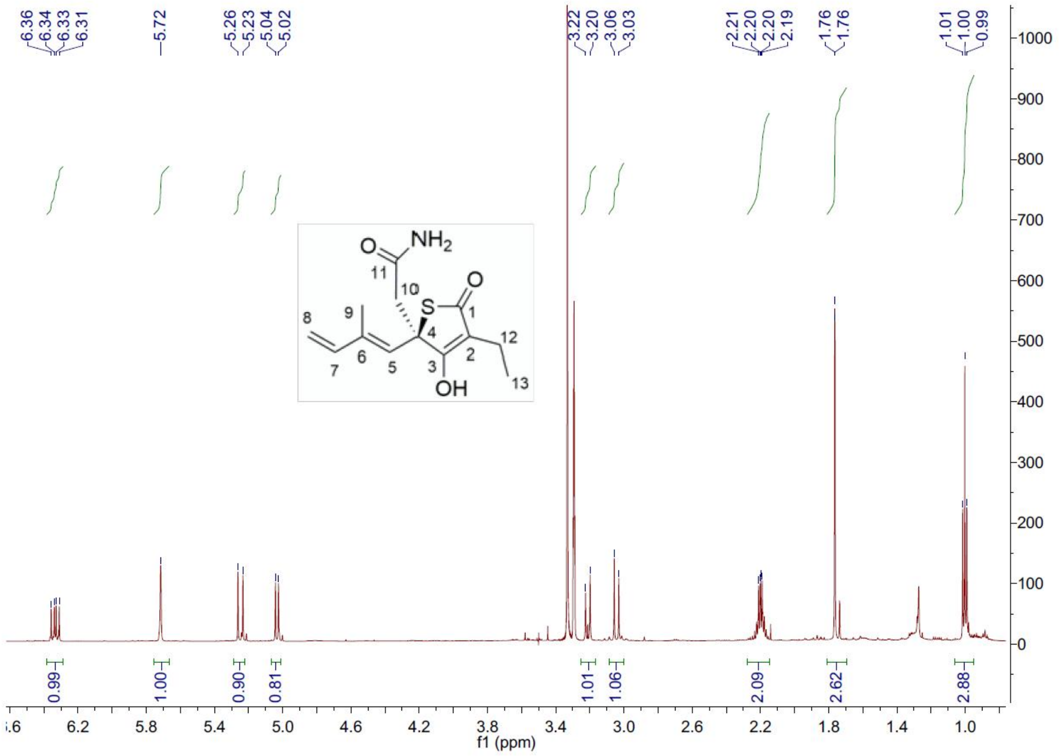

b) ^{13}C-NMR spectrum of Compound 4

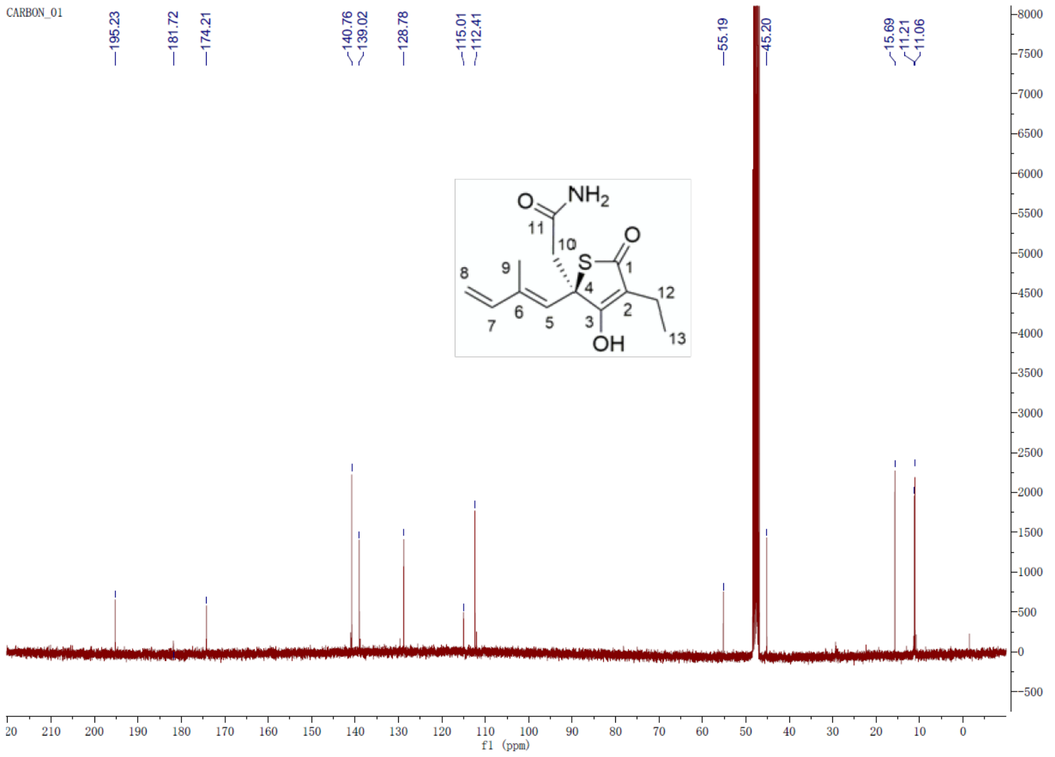

c) ^{1}H-^{1}H COSY spectrum of compound 4

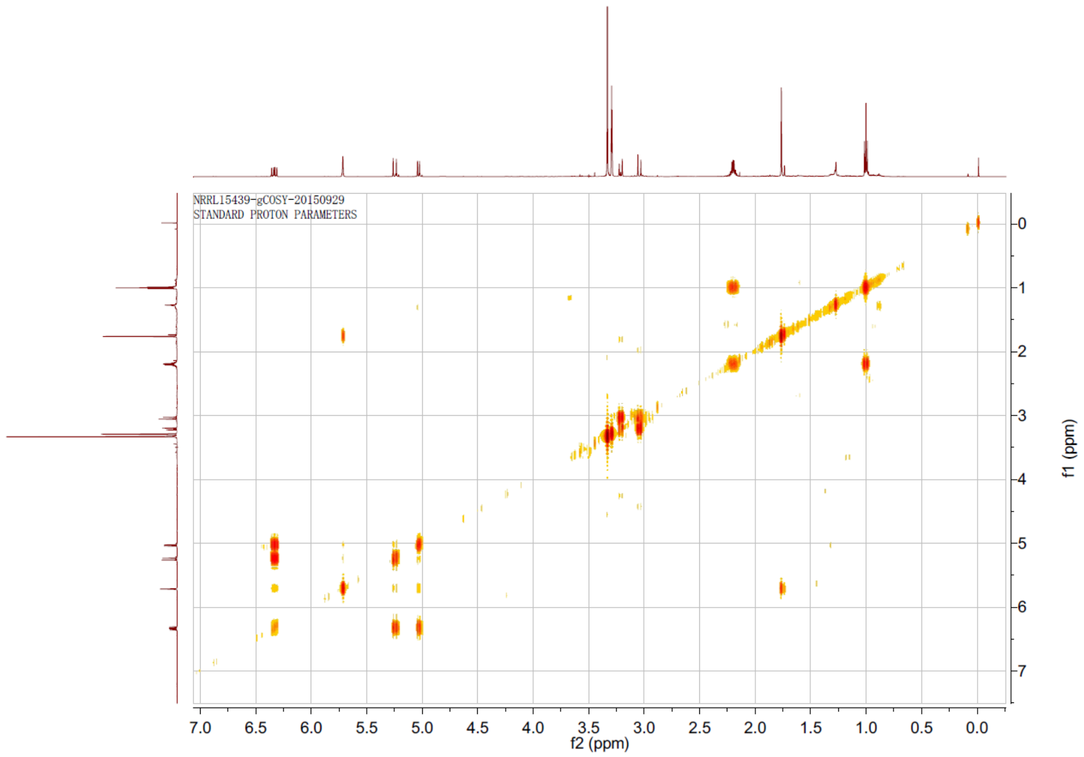

d) HSQC spectrum of compound 4

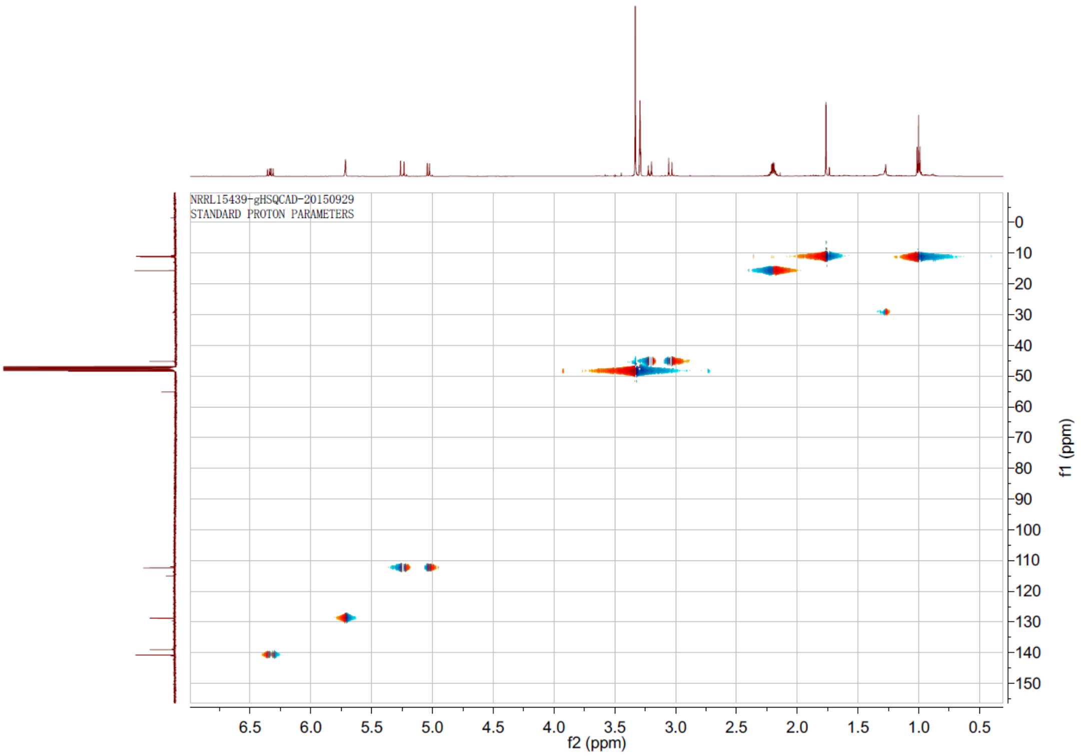

e) HMBC spectrum of compound 4

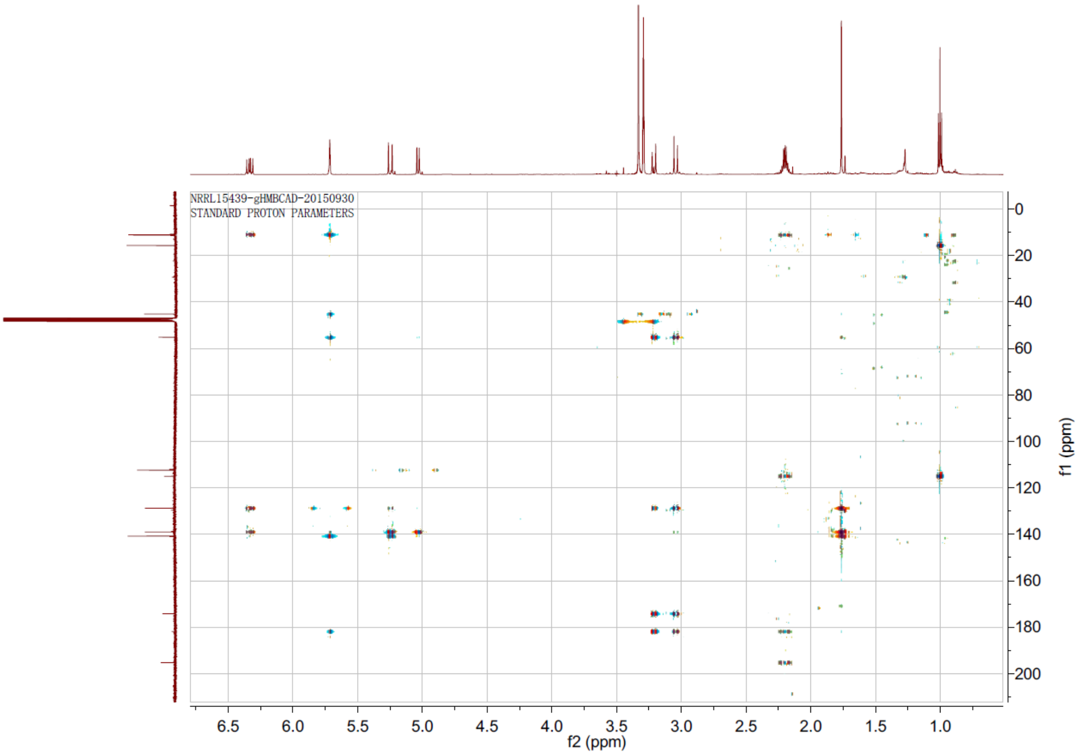

Fig. S2. Selected NMR spectra for compound 4 from *Streptomyces* sp. MG11.

a) ^{1}H-NMR spectrum of compound 4

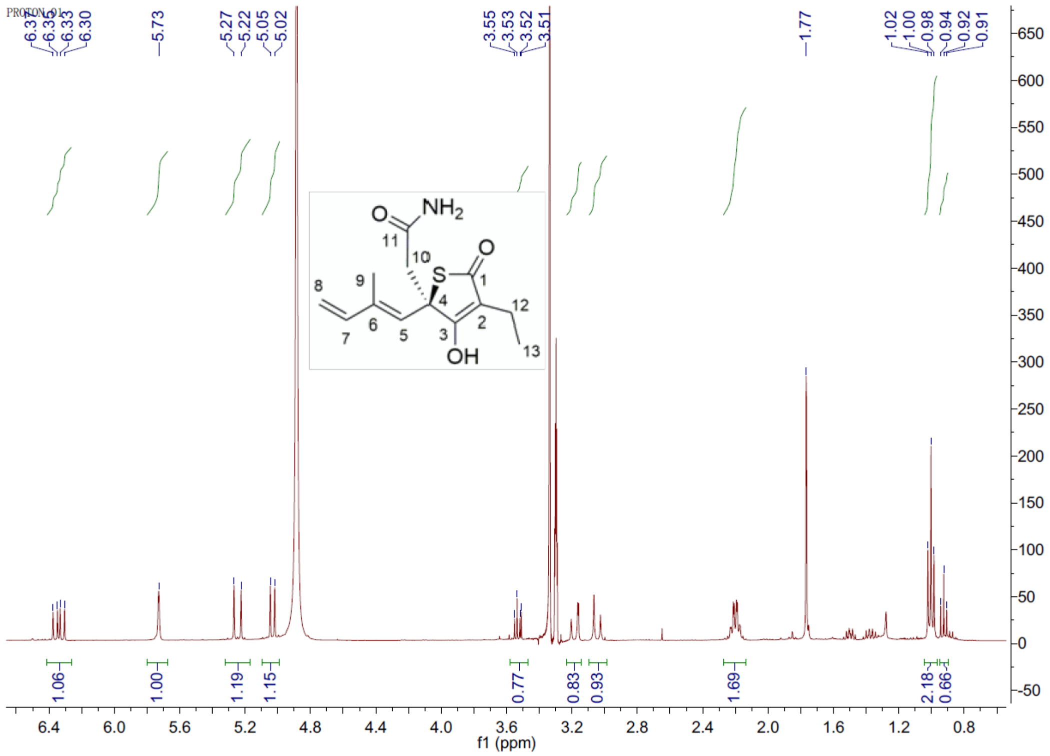

b) ^{13}C-NMR spectrum of compound 4

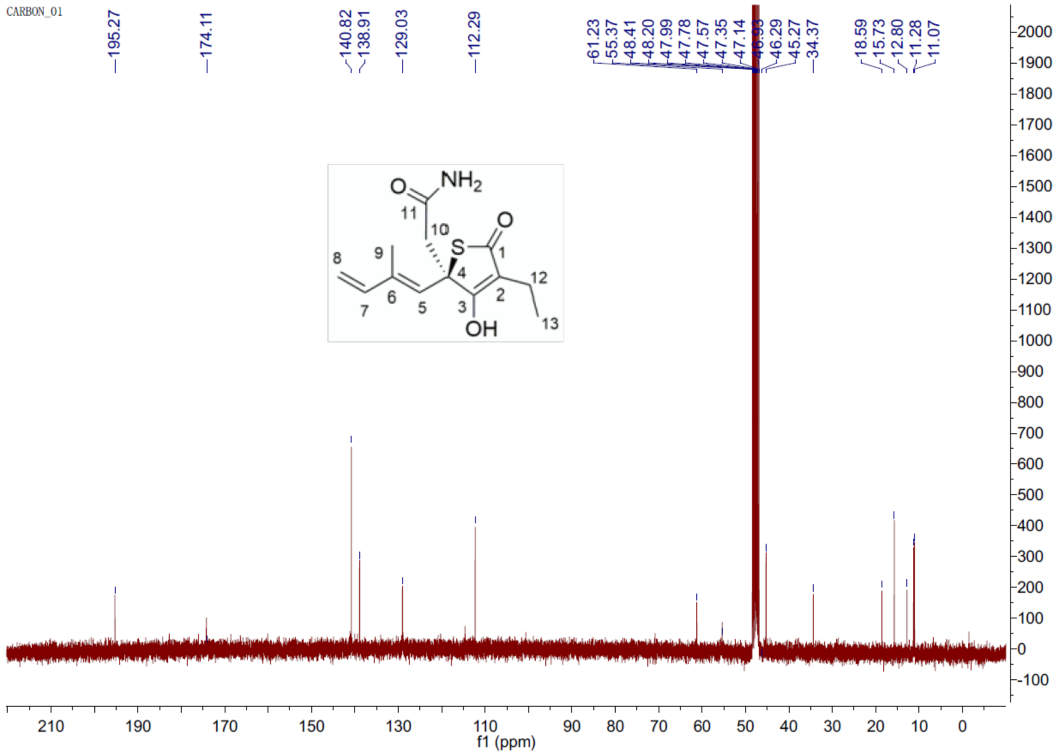

c) ^{1}H-^{1}H COSY spectrum of compound 4

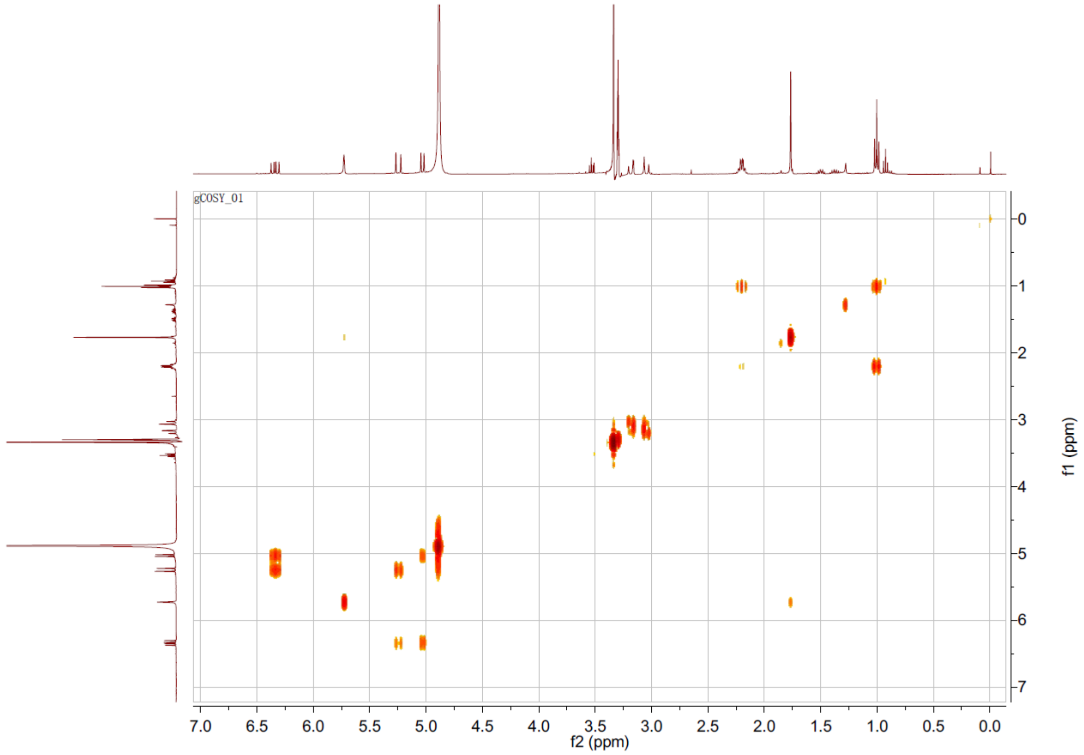

d) HMQC spectrum of compound 4

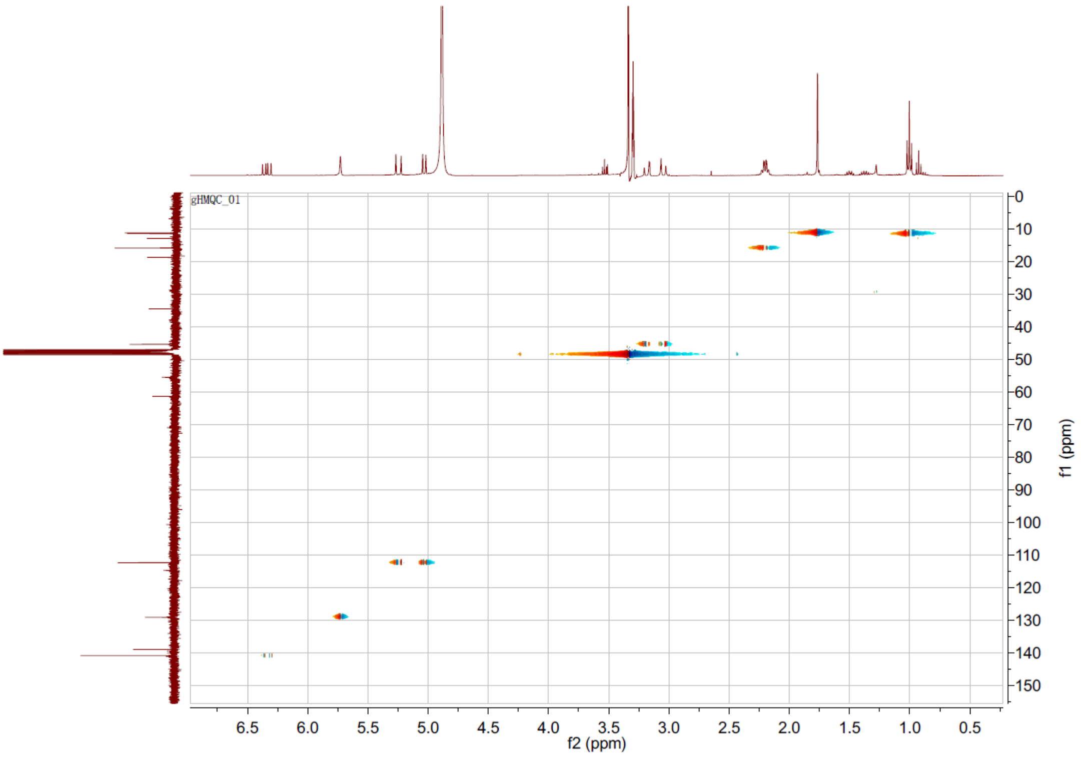

e) HMBC spectrum of compound 4

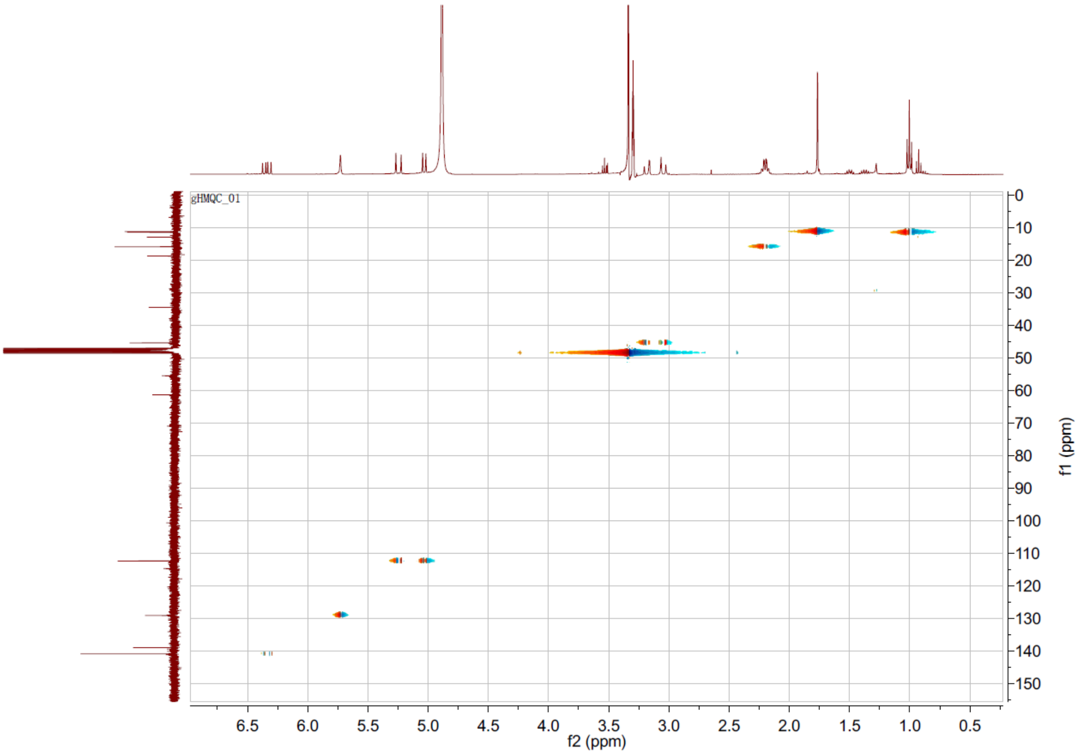

Fig. S3. Chemical structures and key ^{1}H-^{1}H COSY (▬) and HMBC correlations (H⟶C) of compound 4 from *Streptomyces thiolactonus* NRRL 15439 and *Streptomyces* sp. MG11.

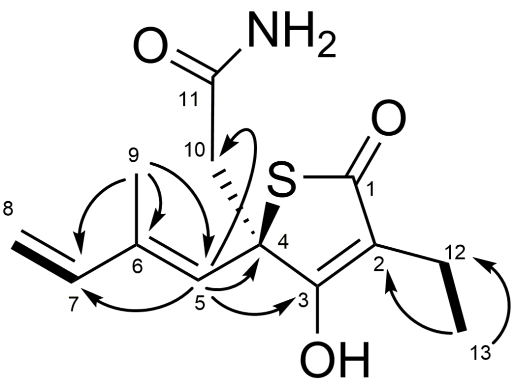

```
                                 620                        720
                                  |                          |
StuB_AT      LAGVRPAAVIGHSQGEVAAACL.........RRVPAKCASHSPVMAELEGS
TueB_AT      LAGVRPAAVVGHSQGEVAAACL.........RRVPAKCASHSPVVAELEGP
SsuB_AT      SVGVEPAAVVGHSQGEVAAACL.........RRVPAKCASHSPVMAELEHP
TlmB_AT      DAGVEPAAVIGHSQGEVAAAHV.........RRVPVRYASHSPAVEPLRAE
```

Fig. S4. Sequence alignment of acyltransferase (AT) domains from thiotetronate PKS enzymes. Specific residues within the active site motifs of an AT domain confer extender unit selectivity for each module. As expected, the AT domain of TlmB in the *tlm* cluster has the specificity motif YASH, which predicts incorporation of methylmalonyl-CoA units in all three cycles of polyketide chain extension. For the *stu, ssu,* and *tue* clusters, the specificity motif is the non-conventional CASH. This could allow the AT domains of StuB, SsuB, and TueB to accept both propionate and butyrate as extension units.

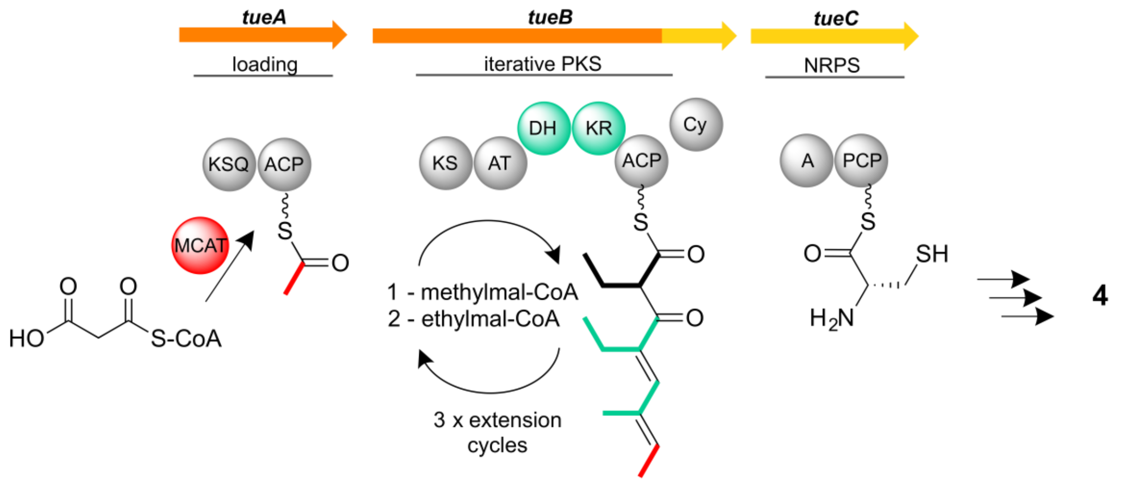

Fig. S5. Biosynthesis of the Tü 3010 tetraketide backbone by the iterative PKS TueB. As in the biosynthesis of the TLM tetraketide (Fig. 2B), the acyltransferase for the loading module is proposed to be contributed by malonyl-CoA: ACP acyltransferase of fatty acid biosynthesis (MCAT). TueB catalyses all three cycles of chain elongation, recruiting methylmalonyl-CoA in the first cycle, and ethylmalonyl-CoA in cycles two and three. As in TlmB, the KR and DH are only active in the first and second cycles, as indicated by the part of the polyketide backbone shown in green. The same domain arrangement and extender unit specificity is seen in the *stu* and *ssu* clusters, except that *ssuC* also encodes for a thioesterase (TE) domain at the C-terminal end of the PCP domain. Sulfur incorporation and thiotetronate ring formation are proposed to follow the same mechanism as shown for TLM in Fig. 5.

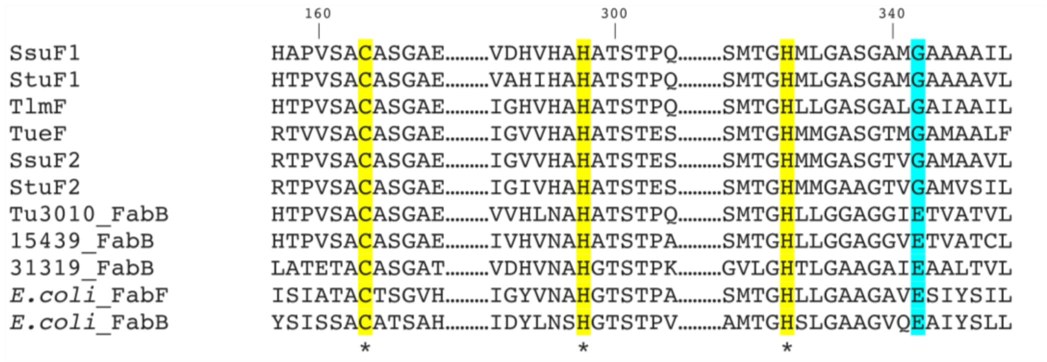

Fig. S6. Sequence alignment of FabB/FabF enzymes from the thiotetronate genetic clusters. The conserved Cys-His-His catalytic triad is highlighted in yellow and marked with asterisks. The Glu342 residue of *E. coli* FabB and FabF, which participates in the active site hydrogen bond network,[11] is replaced in homologues from the thiotetronate clusters by a glycine residue. Samples Tu3010_FabB, 15439_FabB, and 31319_FabB represent FabB homologues from fatty acid synthase (FAS) loci in the strains *S. olivaceus* Tü 3010, *S. thiolactonus* NRRL 15439, and *S. Lentzea* sp. ATCC 31319, respectively. Intriguingly, this E→G mutation is not present in the thiotetronate FAS related FabB homologues.

```
                   110                     240                                                              270
                   |                       |                                                                |
SsuF3          FDVNAVCAGF.........RHFIPHQANGAMLGEVLENLGLPR-ARAHLPVGRHGNTGAAS
StuF3          FDVNAVCAGF.........RHFIPHQANGAMLRDIWGHMGLPR-AHLHMPVARHGNTGAAS
Tu3010_FabH    FDISAGCAGF.........DVFIPHQANMRIIDSMVKTLKLPESVTVARDVETTGNTSAAS
15439_FabH     FDIAAVCTGF.........DKVVLHQANARILAAVAERIDVPL-ERFPANIERVGNTVAAS
31319_FabH     FDVSAVCTGF.........DRVVAHQANARILSLVGQRLSISD-EKVLGNISRVGNTGAAS
E.coli_FabH    FDVAAACAGF.........DWLVPHQANLRIISATAKKLGMSM-DNVVVTLDRHGNTSAAS
                     *                  *                                                                *
```

Fig. S7. Sequence alignment of FabH enzymes from thiotetronate gene clusters. The conserved Cys-His-Asn catalytic triad in *E. coli* 3-oxoacyl-ACP synthase III (FabH) is highlighted in yellow and marked with asterisks. Residues marked in blue are amino acids that have been confirmed as critical to the *E. coli* FabH-ACP interaction.[12] Samples Tu3010_FabH, 15439_FabH, and 31319_FabH represent FabH homologues from fatty acid synthase (FAS) loci in the strains *S. olivaceus* Tü 3010, *S. thiolactonus* NRRL 15439, and *S. Lentzea* sp. ATCC 31319, respectively.

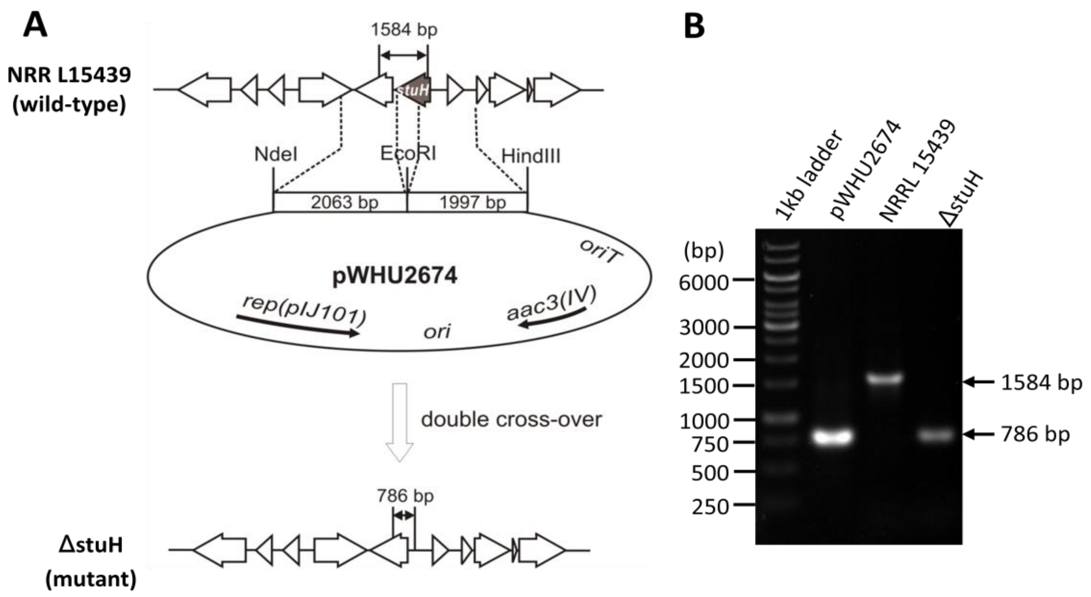

Fig. S8. In-frame deletion of *stuH* in *S. thiolactonus* NRRL 15439. (A) Schematic representation of the in-frame deletion of *stuH.* (B) PCR confirmation of ΔstuH. The arrows indicate the expected size of the fragments from the wild-type and mutant chromosomal DNA, respectively.

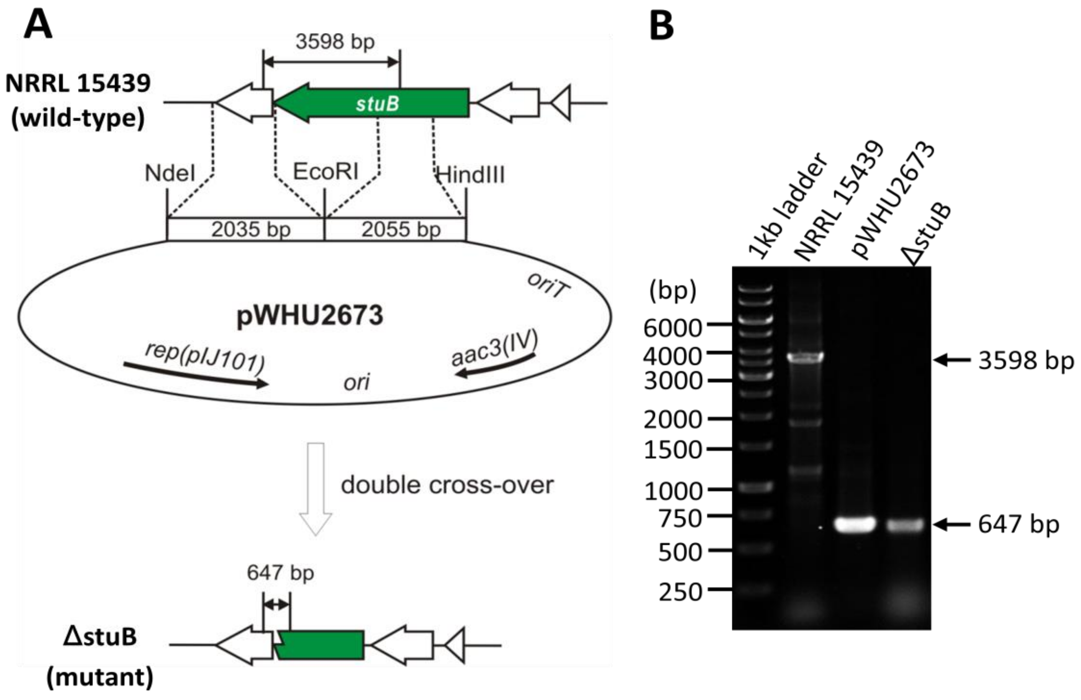

Fig. S9. In-frame deletion of *stuB* in *S. thiolactonus* NRRL 15439. (A) Schematic representation of in-frame deletion of *stuB*. (B) PCR confirmation of ΔstuB. The arrows indicate the expected size of the fragments from the wild-type and mutant chromosomal DNA, respectively.

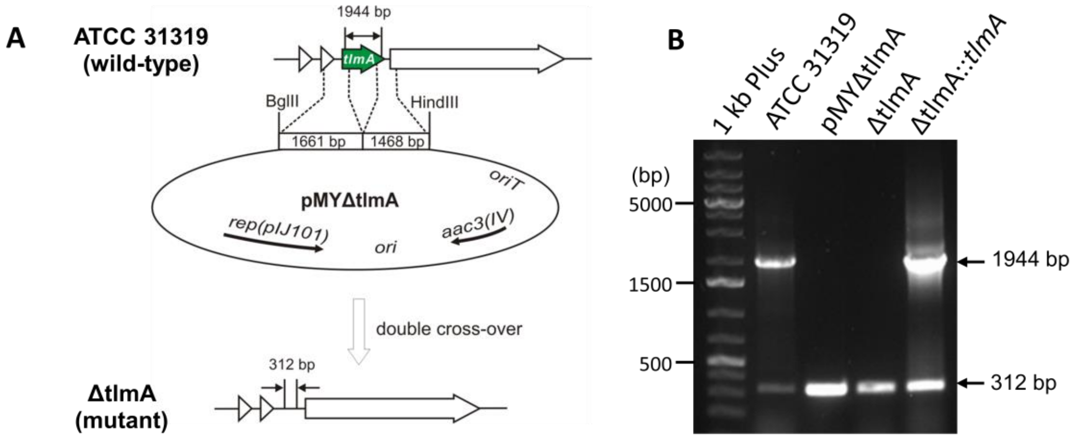

Fig. S10. In-frame deletion of *tlmA* in *Lentzea* sp. ATCC 31319. (A) Schematic representation of the in-frame deletion of *tlmA*. (B) PCR confirmation of ΔtlmA. The arrows indicate the expected size of the fragments from the wild-type and mutant chromosomal DNA, respectively.

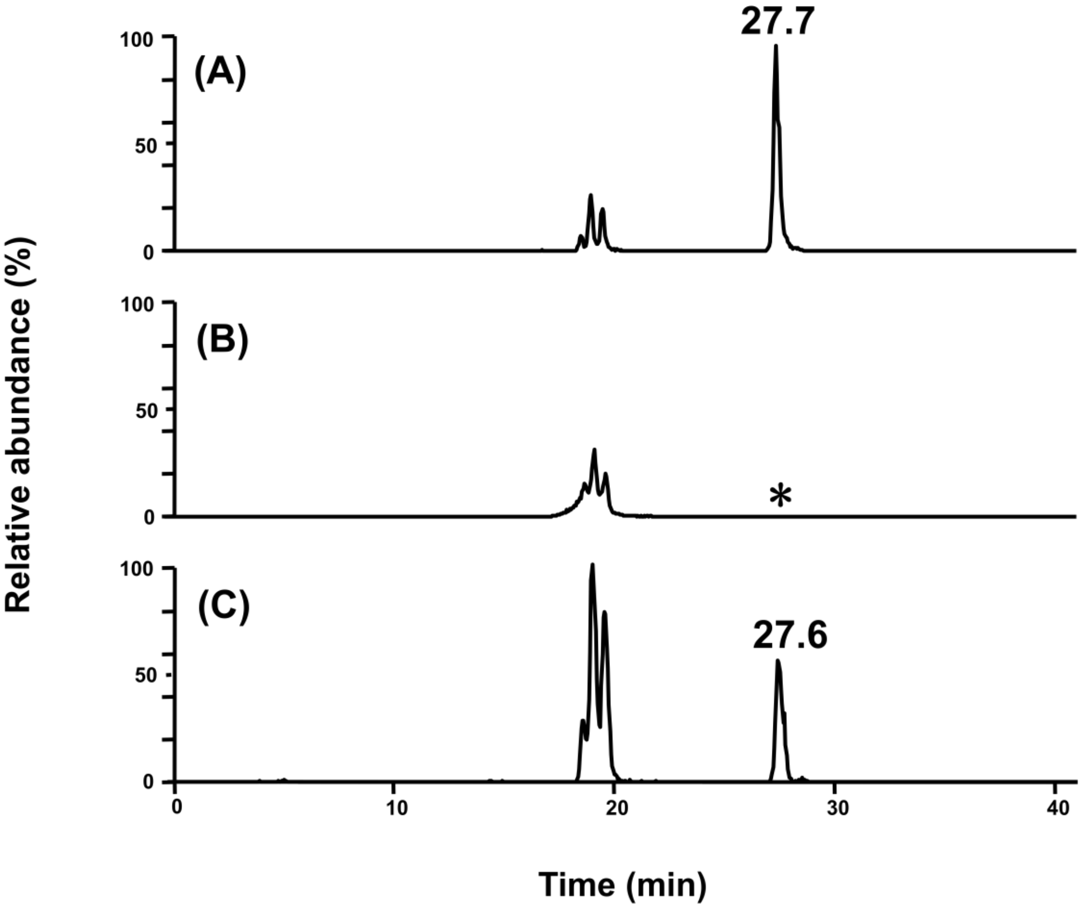

Fig. S11. LC-ESI-MS analysis of thiolactomycin (TLM) from *Lentzea* sp. ATCC 31319, selective ion monitoring of *m/z* 211.2. (A) TLM from wild-type $[M+H]^+$ (*m/z* 211.2) elutes at 27.7 min; (B) *Lentzea* sp. ΔtlmA strain, the asterisk means not detected; and (C) the complementation strain *Lentzea* sp. ΔtlmA::pIB-tlmA. The other peak present, eluting at 19.4 minutes (*m/z* 211.3), has been confirmed to be an artifact, unrelated to thiolactomycin.

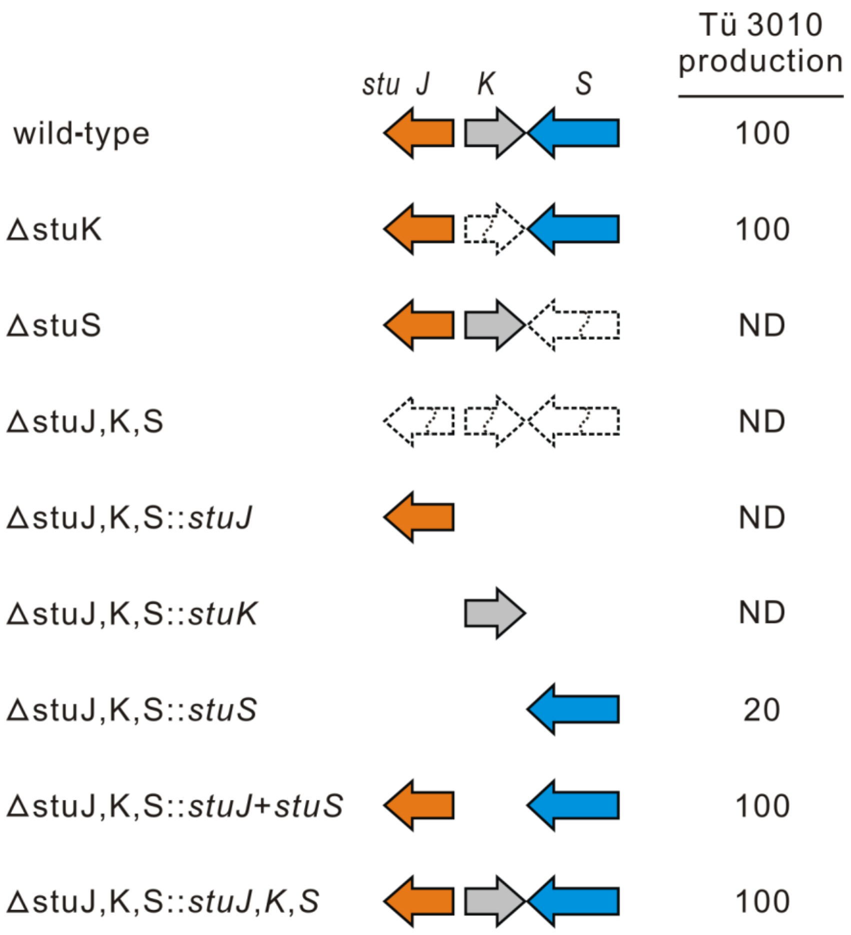

Fig. S12. Genetic analysis of sulfur metabolism genes involved in Tü 3010 biosynthesis. Genes were subjected to either in-frame deletion or gene replacement, and selected mutants were complemented *in trans* using wild-type genes *in vivo*. Genes mutated are shown in dotted lines. *stuS*, NifS-like cysteine desulfurase; *stuJ*, tRNA-specific 2-thiouridylase; *stuK*, *N*-acetylmuramoyl-L-alanine amidase (not involved in Tü 3010 biosynthesis). Production of Tü 3010 is expressed as a percentage of wild-type levels. ND, not detected.

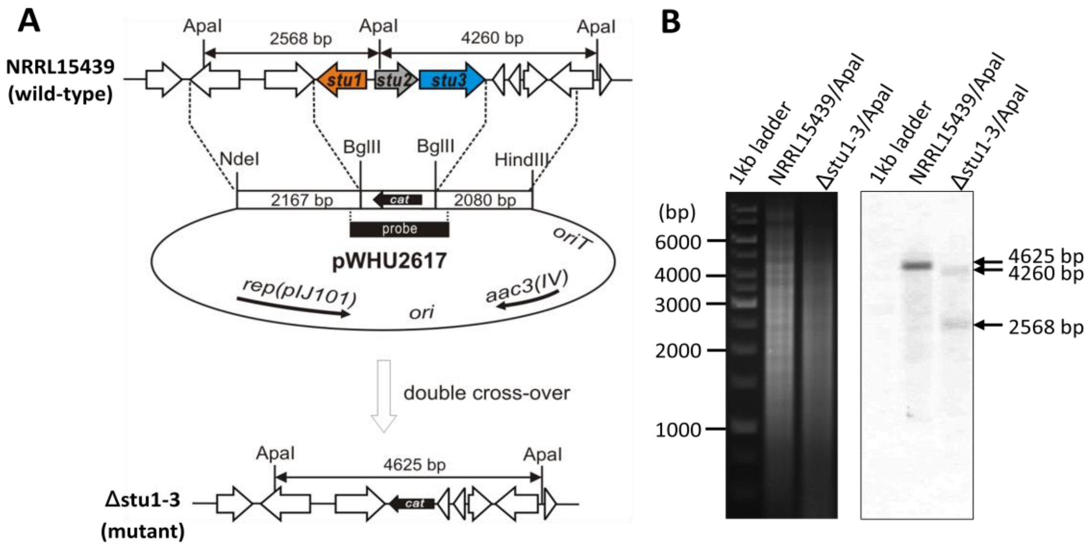

Fig. S13. Replacement of *stuJ,K,S* genes by chloramphenicol resistance gene (*cat*) in *S. thiolactonus* NRRL 15439. (A) Schematic representation of the replacement of *stuJ,K,S* by *cat*. (B) Southern blot confirmation of ΔstuJ,K,S::*cat*. The arrows indicate the expected size of the fragments from the wild-type and mutant chromosomal DNA, respectively.

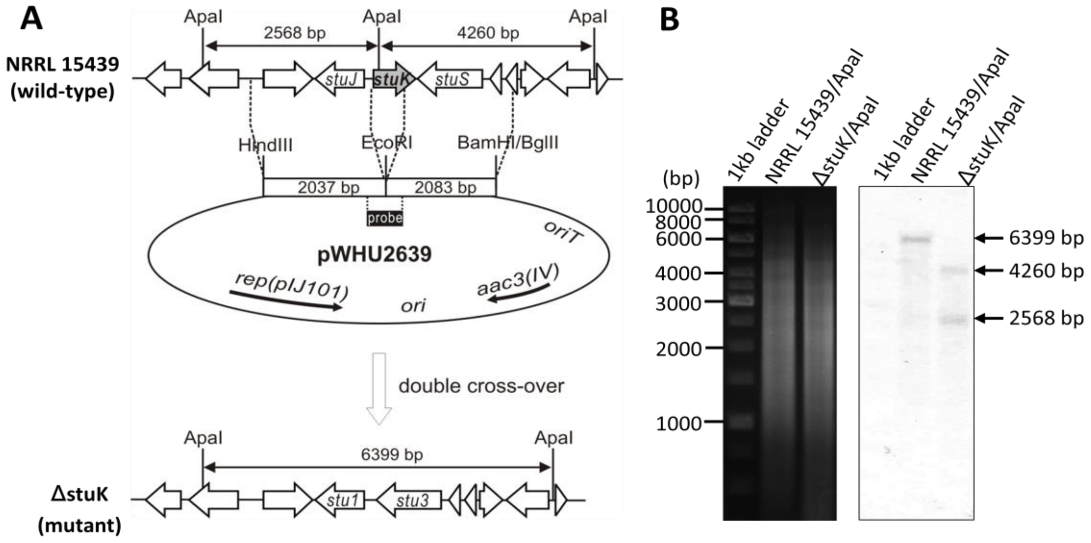

Fig. S14. In-frame deletion of *stuK* in *S. thiolactonus* NRRL 15439. (A) Schematic representation of in-frame deletion of *stuK*. (B) Southern blot confirmation of ΔstuK. The arrows indicate the expected size of the fragments from the wild-type and mutant chromosomal DNA, respectively.

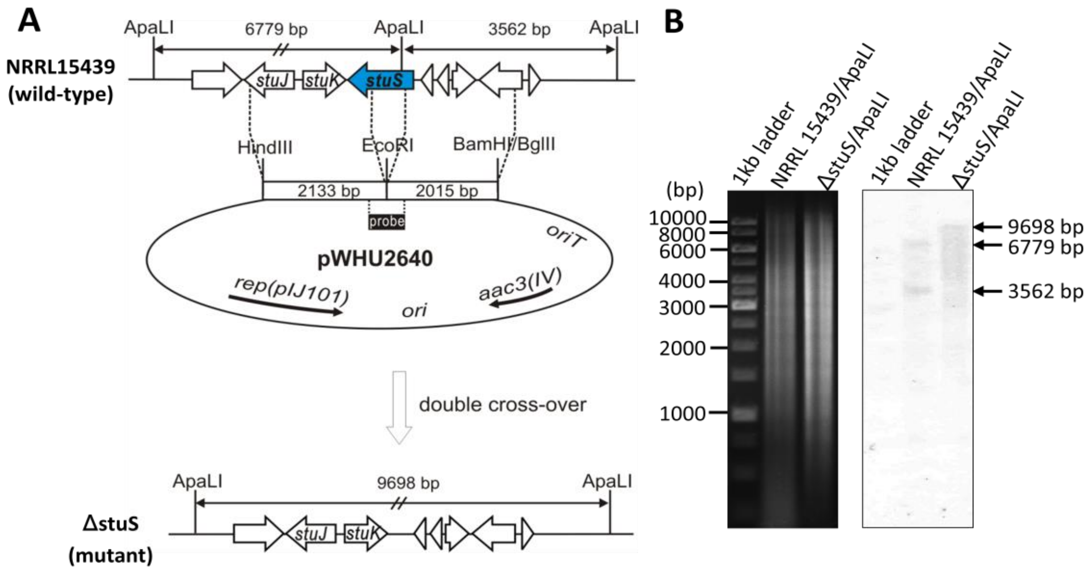

Fig. S15. In-frame deletion of *stuS* in *S. thiolactonus* NRRL 15439. (A) Schematic representation of in-frame deletion of *stuS*. (B) Southern blot confirmation of ∆stuS. The arrows indicate the expected size of the fragments from the wild-type and mutant chromosomal DNA, respectively.

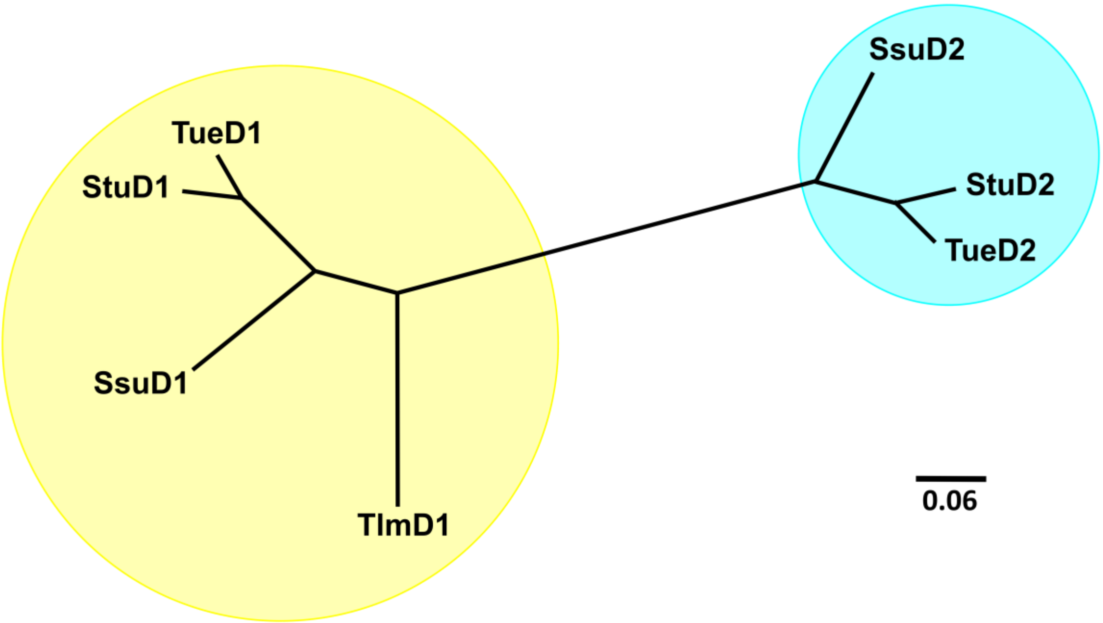

Fig. S16. Phylogram of cytochrome P450 enzymes encoded in thiotetronate biosynthetic gene clusters. Amino acid sequences were aligned with ClustalW2 and the resulting phylogenetic tree was generated using the Neighbor-Joining method without manual adjustment. The phylogenetic tree was rendered using FigTree v1.[13]

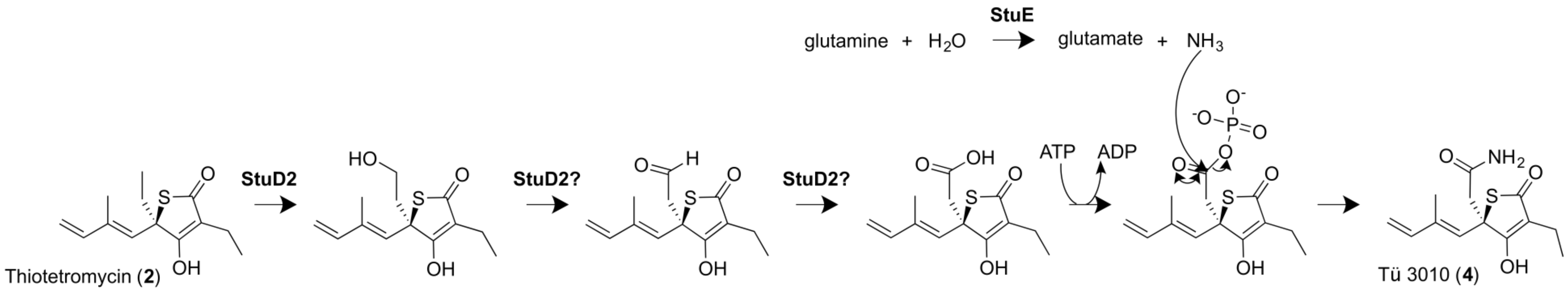

Fig. S17. Proposed mechanism for formation of the carboxamide function of Tü 3010. TLM does not undergo analogous oxidation and the *tlm* cluster accordingly lacks genes encoding close homologues of the enzymes required.

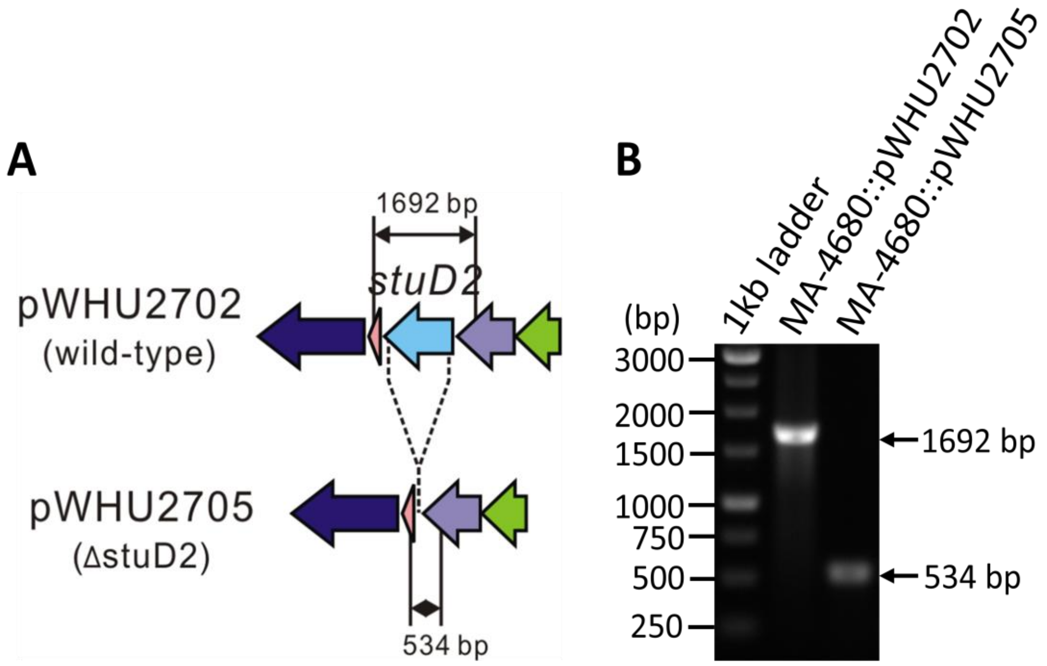

Fig. S18. In-frame deletion of *stuD2* in pWHU2702. (A) Schematic representation of in-frame deletion of *stuD2* in pWHU2702. (B) PCR confirmation of MA-4680::pWHU2705. The arrows indicate the expected size of the fragments from MA-4680::pWHU2702 and MA-4680::pWHU2705 chromosomal DNA, respectively.

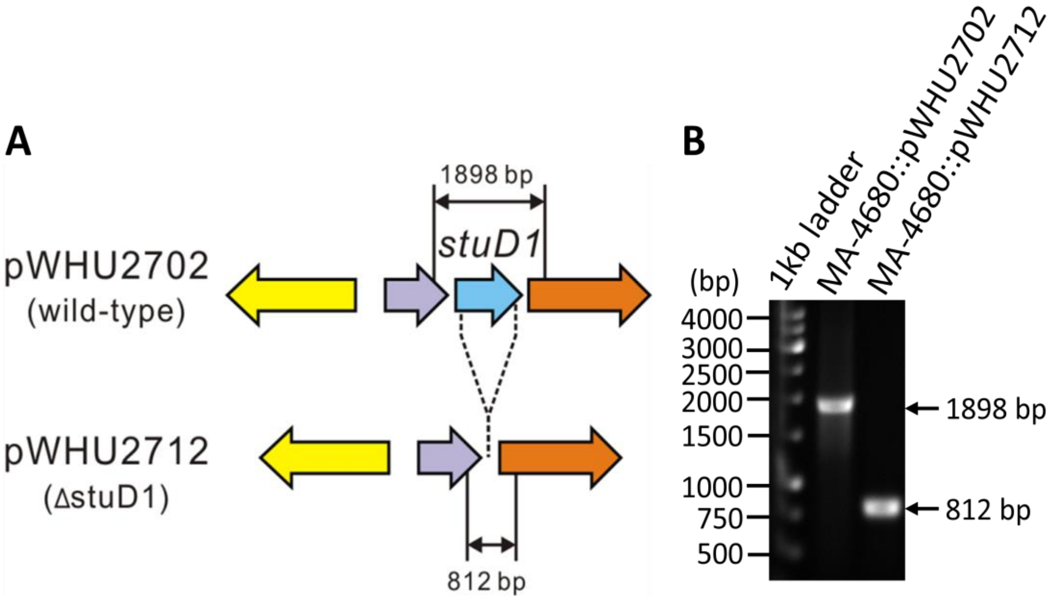

Fig. S19. In-frame deletion of *stuD1* in pWHU2702. (A) Schematic representation of in-frame deletion of *stuD1* in pWHU2702. (B) PCR confirmation of MA-4680::pWHU2712. The arrows indicate the expected size of the fragments from MA-4680::pWHU2702 and MA-4680::pWHU2712 chromosomal DNA, respectively.

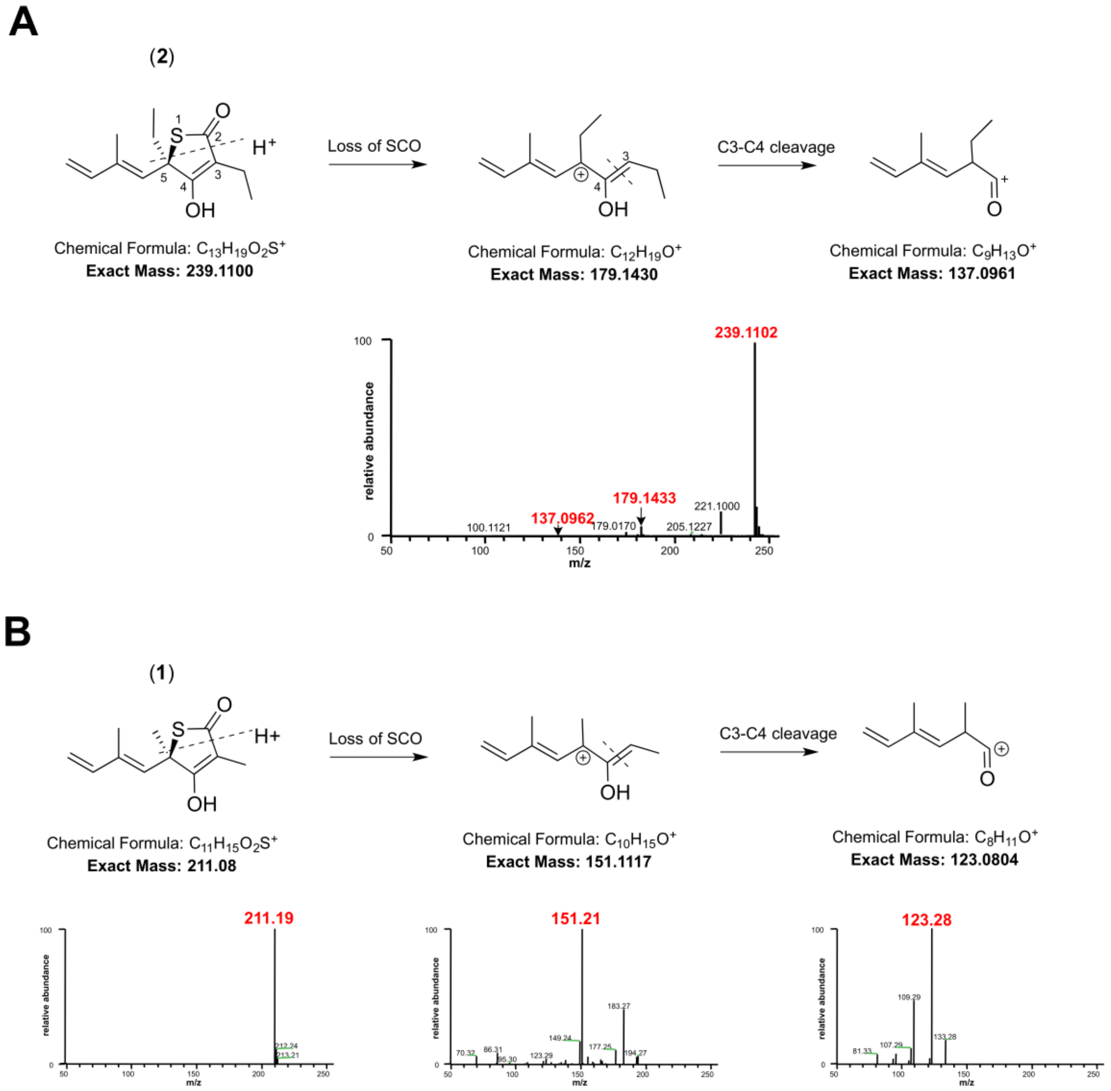

Fig. S20. Fragmentation pathway for (A) thiotetromycin (**2**) and (B) thiolactomycin (**1**) based on MS-MS and MS^3 analysis. A) The high resolution ESI-MS spectra of major ΔstuD2 metabolites (**2**). B) The low resolution ESI-MS spectra of thiolactomycin and major fragments. For both thiotetromycin (**2**) and thiolactomycin (**1**) loss of CO is also a major MS-MS fragment.

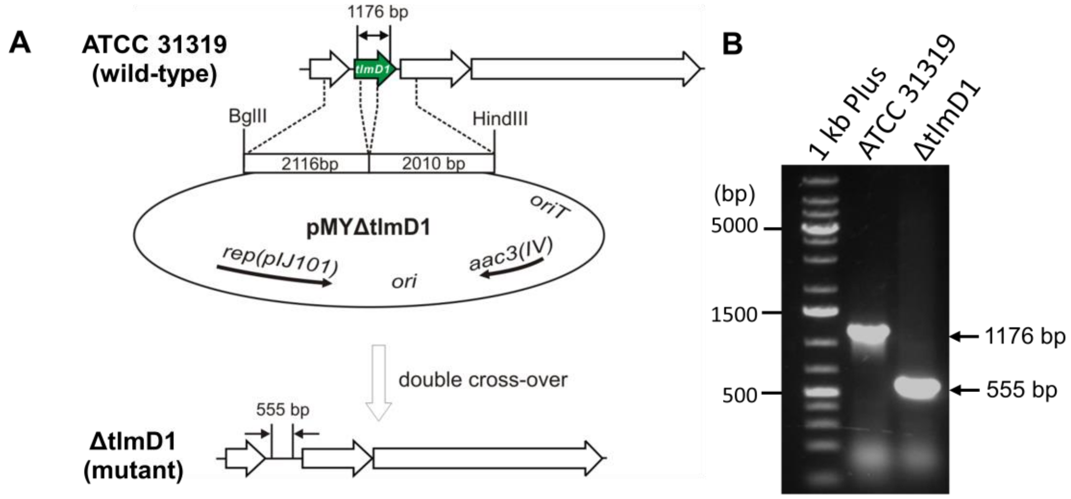

Fig. S21. In-frame deletion of *tlmD1* in *Lentzea* sp. ATCC3139. (A) Schematic representation of the in-frame deletion of *tlmD1*. (B) PCR confirmation of ΔtlmD1. The arrows indicate the expected size of the fragments from the wild-type and mutant chromosomal DNA, respectively.

References

1. T. Kieser, M. J. Bibb, M. J. Buttner, K. F. Chater and D. A. Hopwood, 2000, Practical *Streptomyces* Genetics: A Laboratory Manual (The John Innes Foundation, Norwich, U.K.).
2. C. Rapp, G. Jung, C. Isselhorst-Scharb and H. Zähner, *Liebigs Ann. Chem.*, 1988, **1988**, 1043.
3. L. Li, Z. Lu, X. Xu, J. Wu, Y. Zhang, X. He, T. M. Zabriskie and Z. Deng, *ChemBioChem*, 2008, **9**, 1286.
4. Y. Sun, X. He, J. Liang, X. Zhou and Z. Deng, *Appl. Microbiol. Biotechnol.*, 2009, **82**, 303.
5. D. J. MacNeil, K. M. Gewain, C. L. Ruby, G. Dezeny, P. H. Gibbons and T. MacNeil, *Gene*, 1992, **111**, 61.
6. H. Oishi, T. Noto, H. Sasaki, K. Suzuki, T. Hayashi, H. Okazaki, K. Ando and M. Sawada, *J. Antibiot.*, 1982, **35**, 391.
7. L. A. Dolak, T. M. Castle, S. E. Truesdell and O. K. Sebek, *J. Antibiot*., 1986, **39**, 26.
8. R. W. Burg, B. M. Miller, E. E. Baker, J. Birnbaum, S. A. Currie, R. Hartman, Y. L Kong, R. L. Monaghan, G. Olson, I. Putter, J. B. Tunac, H. Wallick, E. O. Stapley, R. Oiwa and S. Omura, *Antimicrob. Agents Chemother.*, 1979, **15**, 361.
9. J. Sambrook, E. F. Fritsch and T. Maniatis, 1989, Molecular Cloning: A Laboratory Manual (Cold Spring Harbor Lab. Press, Plainview, NY), 2nd Ed.
10. C. J. Wilkinson, Z. A. Hughes-Thomas, C. J. Martin, I. Böhm, T. Mironenko, M. Deacon, M. Wheatcroft, G. Wirtz, J. Staunton and P. F. Leadlay, *J. Mol. Microbiol. Biotechnol.*, 2002, **4**, 417.
11. S. White, J. Zheng, Y. Zhang and C. O. Rock, *Annu. Rev. Biochem.*, 2005, **74**, 791.

12. Y. M. Zhang, M. S. Rao, R. J. Heath, A. C. Price, A. J. Olson, C. O. Rock and S. W. White, *J. Biol. Chem.*, 2001, **276**, 8231.
13. A. Rambaut, 2009, FigTree version 1.3.1 [computer program] http://tree.bio.ed.ac.uk.
